# A Flexible Smart Healthcare Platform Conjugated with Artificial Epidermis Assembled by Three-Dimensionally Conductive MOF Network for Gas and Pressure Sensing

**DOI:** 10.1007/s40820-024-01548-5

**Published:** 2024-10-25

**Authors:** Qingqing Zhou, Qihang Ding, Zixun Geng, Chencheng Hu, Long Yang, Zitong Kan, Biao Dong, Miae Won, Hongwei Song, Lin Xu, Jong Seung Kim

**Affiliations:** 1 State Key Laboratory of Integrated Optoelectronics, College of Electronic Science and Engineering, Jilin University, Changchun, 130012 People’s Republic of China; 2 Department of Chemistry, Korea University, Seoul, 02841 Republic of Korea; 3 TheranoChem Incorporation, Seoul, 02856 Republic of Korea

**Keywords:** Ti_3_C_2_T_*x*_@Cu_3_(HHTP)_2_ composites, NO_2_/pressure flexible sensors, Health-monitoring, Machine learning

## Abstract

The rising flexible and intelligent electronics greatly facilitate the noninvasive and timely tracking of physiological information in telemedicine healthcare. Meticulously building bionic-sensitive moieties is vital for designing efficient electronic skin with advanced cognitive functionalities to pluralistically capture external stimuli. However, realistic mimesis, both in the skin’s three-dimensional interlocked hierarchical structures and synchronous encoding multistimuli information capacities, remains a challenging yet vital need for simplifying the design of flexible logic circuits. Herein, we construct an artificial epidermal device by in situ growing Cu_3_(HHTP)_2_ particles onto the hollow spherical Ti_3_C_2_T_*x*_ surface, aiming to concurrently emulate the spinous and granular layers of the skin’s epidermis. The bionic Ti_3_C_2_T_*x*_@Cu_3_(HHTP)_2_ exhibits independent NO_2_ and pressure response, as well as novel functionalities such as acoustic signature perception and Morse code-encrypted message communication. Ultimately, a wearable alarming system with a mobile application terminal is self-developed by integrating the bimodular senor into flexible printed circuits. This system can assess risk factors related with asthmatic, such as stimulation of external NO_2_ gas, abnormal expiratory behavior and exertion degrees of fingers, achieving a recognition accuracy of 97.6% as assisted by a machine learning algorithm. Our work provides a feasible routine to develop intelligent multifunctional healthcare equipment for burgeoning transformative telemedicine diagnosis.

**Supplementary Information:**

The online version contains supplementary material available at 10.1007/s40820-024-01548-5.

## Highlights

- A smart wearable alarming system integrated artificial epidermal device for pluralistically identifying asthmatic attack risk factors, achieving a 97.6% classification accuracy as assisted by machine learning algorithm.
- A meticulous mimicry both in the advanced structural attributes and encoding information abilities of the skin was adopted to design a novel artificial epidermal device by integrating conductive Cu_3_(HHTP)_2_ coupled with spherical Ti_3_C_2_T_*x*_.
- The bioinspired Ti_3_C_2_T_*x*_@Cu_3_(HHTP)_2_ sensors can independently perceive NO_2_ gas and pressure-triggered stimuli.

**Supplementary Information:**

The online version contains supplementary material available at 10.1007/s40820-024-01548-5.

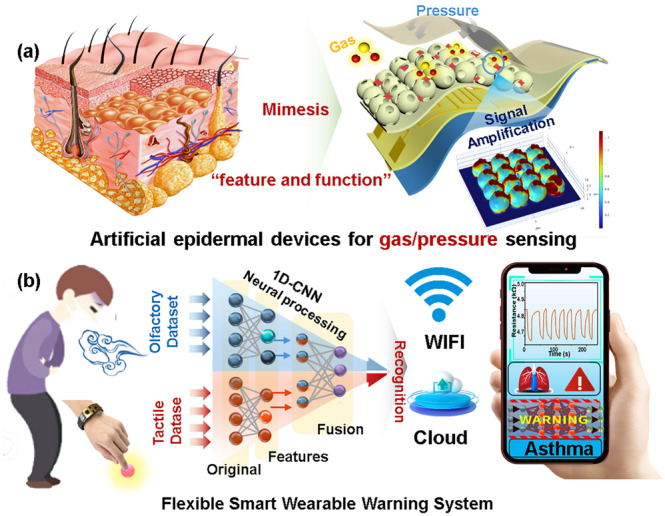

## Introduction

Wearable electronics, characterized by their intelligence, have perpetually exhibited astounding potential in the realms of telemedicine and human–machine interaction (HMI) [1]. This vast potential stems from groundbreaking advancements in sensory and artificial intelligence technologies [2, 3]. One critical application of wearable sensors lies in providing efficient, technically advanced means for unobtrusive, continuous, and real-time monitoring of chronic diseases such as asthma, diabetes, and Parkinson’s [4, 5]. Specifically, by on-site analysis of human sweat, pulse, and respiratory behavior, wearable sensors can assist in establishing electronic databases for individual health assessment [6]. However, the daunting task of assessing the early warning signs of an asthma attack as well as tracing coordination of human–computer interaction creates stringent performance criteria for existing sensors. These sensory devices also require excellent flexibility, elasticity, and resilience to accommodate large deformation [7]. Moreover, the emergence of multi-responsive flexible electronics has doubtlessly dictated the significant potential for HMI, signifying the advent of a revolutionary era driven by an authentic, enriched interactive experience [8, 9]. Consequently, the development and breakthroughs in the realm of multifunctional devices characterized by enhanced sensory capabilities become paramount. Such devices, capable of processing and distilling complex information, become invaluable for portable health monitoring and clinical diagnostics.

As the most expansive organ of the integumentary system, the human skin can interact and communicate diverse external stimuli to exteroceptors, thereby inducing a variety of biopotential impulses depending on the nature of the mechanical stimulus. Drawing inspiration from the advanced structural attributes and signal processing abilities of the skin allows for a visionary model for designing individual bionic devices [10]. Because the skin typically encodes information through the highly compact, parallel, and reliable mode of operation [11], it provides a blueprint to simplify the interaction channels between humans and machines. In fact, the epidermis, being the outermost layer of the skin, consists of microspheres with interleaved and interlocked structures [12]. Learning from the skin’s distinct three-dimensional (3D) topological interconnection architecture, scientists have meticulously designed a series of electronic skin (e-skin) with 3D interlocked hierarchical structures. This includes components like micro-pyramids, microspheres, and sea urchin-like microcapsules engineered to enhance the perception of specific stimuli [13–15]. On the one hand, e-skin boasts an impressive elastic modulus, thereby allowing it to support a broad spectrum of compressive deformation distributions [16]. Further, the e-skin element’s close-contact spherical tip provides an outstanding electromechanical transduction capability coupled with robust mechanical resilience. These unique attributes not only focus and amplify local stress but also enhance sensitivity even in a wide working window [12].

Presently, most artificial e-skin with interconnected structures predominantly focus on simultaneously sensing pressure, strain, and temperature changes [17]. Meanwhile, the other vital gas environment and physiological parameters, such as and hazardous gas and abnormal breathing patterns, which are significant indicators for evaluating human-health status, usually get overlooked [18]. For example, the NO_2_ can act as causes to induce and aggravate asthma disease [19]. Actually, the synchronous integration of sensors recognizing gases and pressure enables the development of more precise and sophisticated methods for screening trace hazardous gas molecules and detecting bodily tremors indicative of early-stage diseases [20]. In particular, the unique layout of 3D interlocked hierarchical structures of e-skin is highly advantageous for facilitating gas adsorption and diffusion. It is expected to efficiently and promptly transform gas stimuli into electrical impulses. However, most current e-skin models exhibit similar or even identical resistance transition mechanisms in response to gas or pressure stimuli [21]. This similarity often leads to mixed electrical outputs or, regretfully, severe signal interference or cross talk. As such, there exists an imperious need to develop an independent, multifunctional sensor capable of providing interference-free response signals and high-precision recognition of stimuli through a streamlined and effective process.

**Fig. 1:**
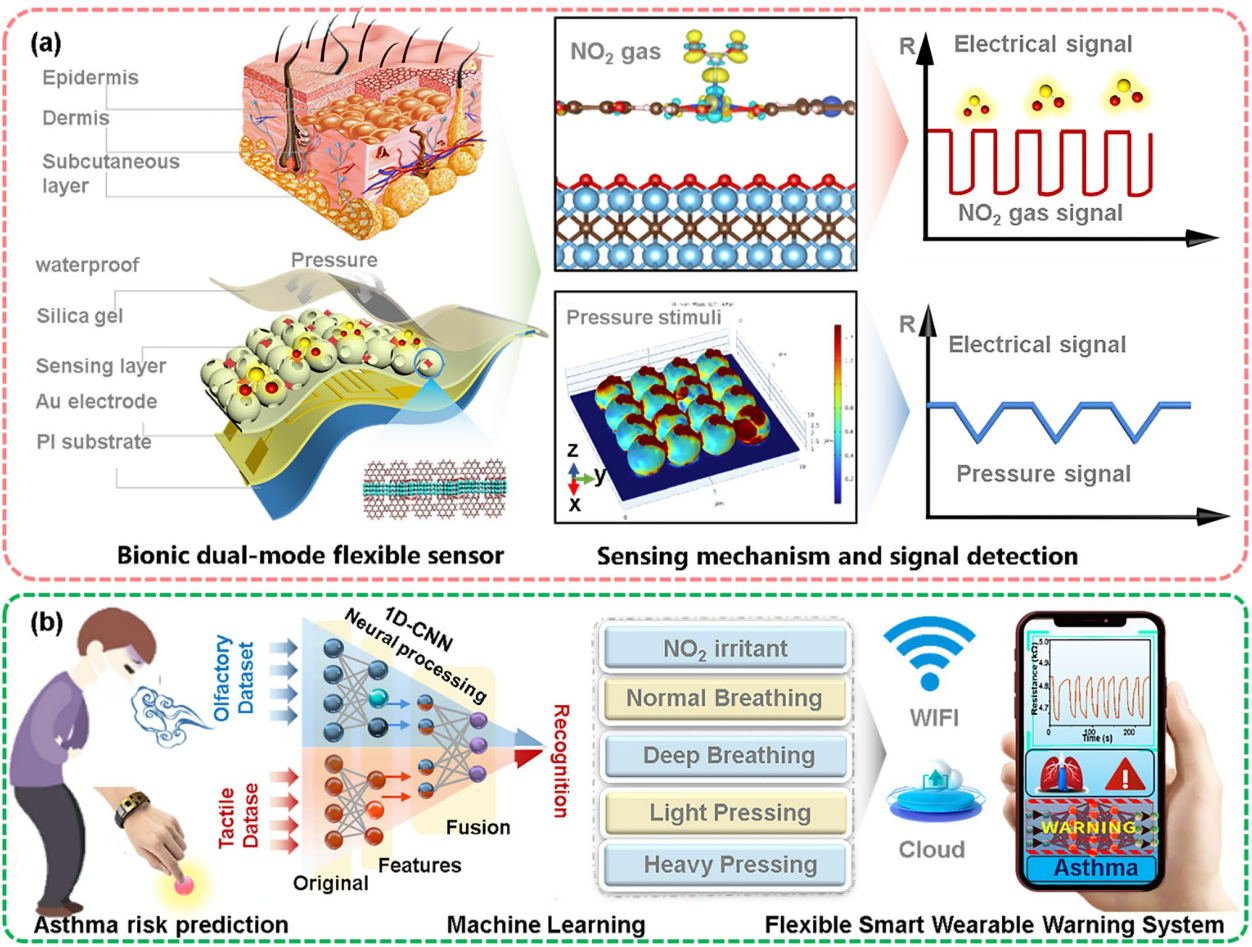
Conceptual design of this study. **a, b** Inspired by the biological tactile sensory structure and neuromorphic system, a flexible smart wearable alarming system with a multifunctional bionic sensor is developed for wireless monitoring and differentiating for the asthmas patients’ physiological signals, as assisted by a machine learning algorithm based on 1-D CNNs

In this work, drawing inspiration from the 3D interlocking hierarchical structures of skin, we designed artificial epidermal device with multi-sensory capabilities to nimbly capture the gas and pressure bioelectrical signals. Herein, the gas-sensitive Cu_3_(HHTP)_2_ is in situ hetero-assembled on a Ti_3_C_2_T_*x*_ interface replicating the hollow spheroidal architecture of the skin, consequently mimicking both the spinous and granular layers of the skin’s epidermis (Fig. 1a). Resultantly, a biomimetic Ti_3_C_2_T_*x*_@Cu_3_(HHTP)_2_ composite is prepared, with Cu_3_(HHTP)_2_ particles responding to external NO_2_ stimuli and a 3D cross-linked network composed of hollow Ti_3_C_2_T_*x*_ spheres controlling the pressure sensing. Thanks to the optimized synergistic effect, enhanced signal transduction capacities, and strong hydrophobic features, the dual-mode sensor demonstrates robust responses to gas and/or pressure stimuli, even in human breathing and sweat-prone skin areas. In addition, novel functionalities such as acoustic signature perception and Morse code-encrypted message communication are endowed into the dual-mode sensor. The developed sensor is further integrated into a flexible printed circuit for wirelessly real-time assessment of risk factors related with asthmatic (Fig. 1b), achieving an impressive 97.6% classification accuracy as assisted by a machine learning algorithm based on 1-D convolutional neural networks (CNNs). This work offers an innovative pathway for building an artificial system for signal identification and transmission, which has potential usage for personal health diagnostics within the realm of telemedicine.

## Experimental Captions

### Fabrication and Measurement of Various Sensors

To prepare the gas sensor, 150 mg of different Ti_3_C_2_T_*x*_, Cu_3_(HHTP)_2_ and Ti_3_C_2_T_*x*_@Cu_3_(HHTP)_2_ samples are weighed separately by an analytical balance, blended with 2 mL ethanol, and then ground into a slurry in a mortar. The slurry is then evenly coated on a commercial flexible polyimide (PI) substrate with interdigital electrodes. The chemiresistive response of all sensors is defined as* (∆R/R*_*0*_*)* × 100%, where *R*_*0*_ and *R*_*g*_ are the electrical resistance of sensor in the presence of air and analyzed gases, respectively. The sensitivity is regarded as the slope of the calibration curve. The response/recovery time was defined as the time duration from the initial resistance value to 90% of the final equilibrium value. The gas sensing measurements were assessed by Fluke-8846A. The chemiresistive response tests of the sensor were performed at 25 °C. The adopted standard analyte vapors including NH_3_, H_2_S, CH_3_COCH_3_, C_6_H_7_, HCHO, CH_3_OH, and C_2_H_5_OH are fabricated via the static volumetric method.

### Preparation of the Flexible Piezoresistive Sensor

The optimal Ti_3_C_2_T_*x*_@Cu_3_(HHTP)_2_ solution was directed dripped onto the commercial flexible PI substrate with Au electrodes and then dried naturally. Cu copper wire was soldered to both sides of the Au electrode, and then, the silica gel is in situ encapsulated on the top of the Au electrode to construct the flexible piezoresistive sensor.

### Development of Flexible Smart Wearable Alarming System

To be specific, the flexible smart wearable alarming system integrates with a dual-mode bionic sensor, analog-to-digital converter (ADC), Wi-Fi wireless module, ESP32 chip, micro-USB port, and power supply. ESP32 module was conducted to read the resistance data of the flexible dual-mode sensor and upload it to the Alibaba Cloud IoT by utilizing a divider circuit structure and then converted to digital domain via an analog-to-digital converter (ADC). Subsequently, the cloud allows access to save the resistance data acquired from the dual-mode flexible sensor in the server database, finally upload it to the smartphones or computers terminal. The smart mobile terminal utilizes a self-developed supporting application to process and display the forwarded data from the cloud. Finally, the flexible smart wearable alarming system was developed to the acquisition of human physiological signals. Specifically, two copper wires are connected to both ends of the flexible sensor, and then, the other ends of the copper wire are further welded into the flexible printed circuit. Moreover, the sensor is connected on the wrist for detection of NO_2_ atmosphere, easier to trigger asthma disease. All procedures of the investigation in human subjects were performed in compliance with recognized standards of the Ethical Review Methods for Biomedical Research involving Humans adopted by the National Health and Family Planning Commission of the People’s Republic of China. This research includes some sensor experiments about breathing detection and conceptual demonstration on human research participants. All experiments involving human participants were done only with the authors, and informed written consent was obtained from the participants.

## Results and Discussion

### Preparation and Characterization of Ti_3_C_2_T_***x***_@Cu_3_(HHTP)_2_ Composite

**Fig. 2:**
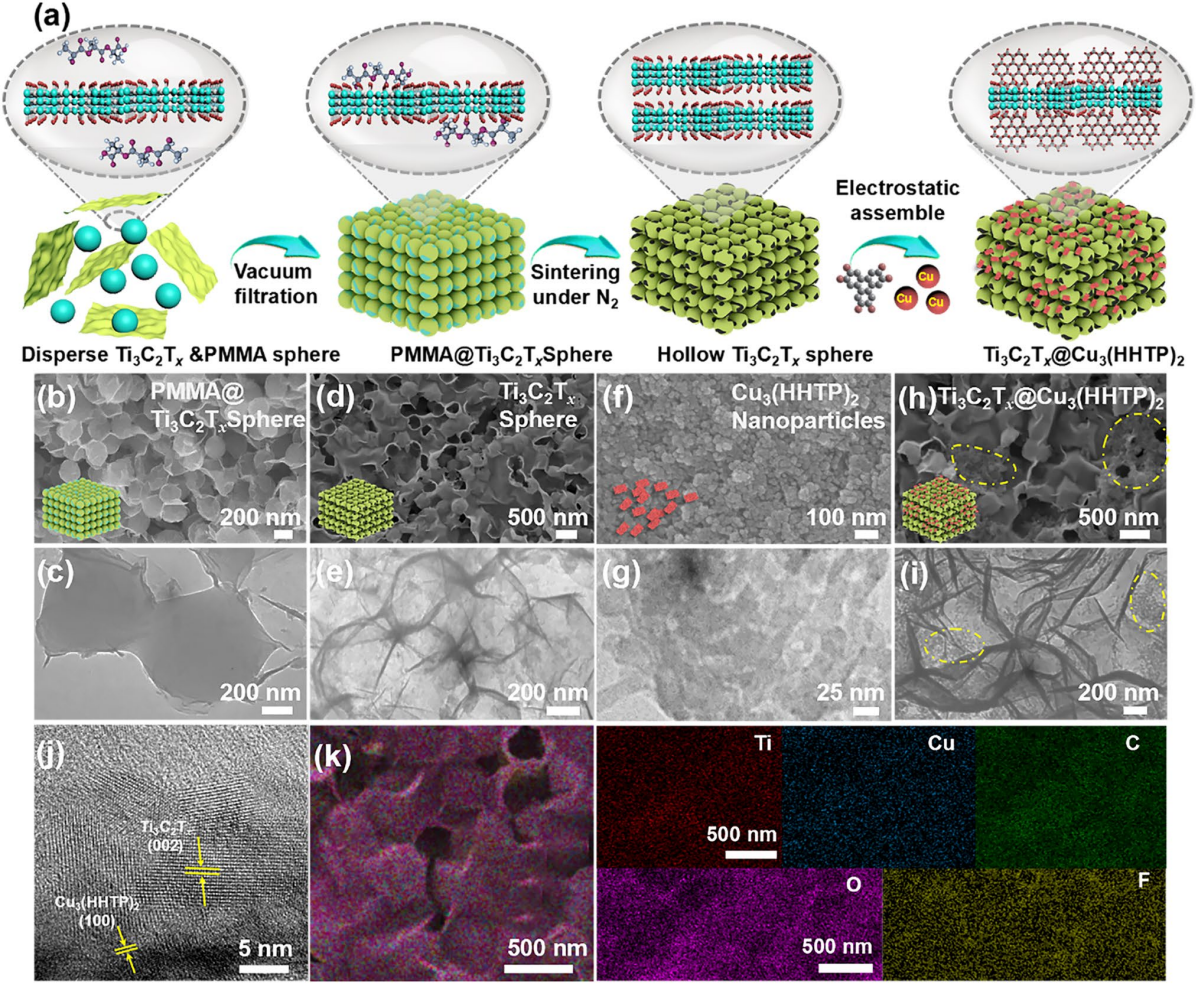
**a** Schematic illustration of the synthetic process of the Ti_3_C_2_T_*x*_ foam, Cu_3_(HHTP)_2_ particles and Ti_3_C_2_T_*x*_@Cu_3_(HHTP)_2_ composite; **b** SEM and **c** TEM images of the PMMA@Ti_3_C_2_T_*x*_ spheres, respectively; **d** SEM and **e** TEM images of the hollow Ti_3_C_2_T_*x*_ foam; **f** SEM and **g** TEM images of Cu_3_(HHTP)_2_ particles; and **h** SEM, **i** TEM, **j** HRTEM images and **k** EDX mapping images of the Ti_3_C_2_T_*x*_@Cu_3_(HHTP)_2_ composite

The synthetic procedure of the biomimetic hierarchical Ti_3_C_2_T_*x*_@Cu_3_(HHTP)_2_ composite is schematically demonstrated in Fig. 2a. Firstly, a few-layered Ti_3_C_2_T_*x*_ MXene flakes with large planar sizes are fabricated by selectively etching the parental Ti_3_AlC_2_ with LiF/HCl acid mixture (Fig. S1). As driven by the strong hydrogen bonds and van der Waals force, the Ti_3_C_2_T_*x*_ flakes can spontaneously wrap and self-assemble onto the PMMA spheres to form the uniform PMMA@Ti_3_C_2_T_*x*_ spheres (Fig. 2b, c) [22]. As shown in Figs. 2c and S2, in contrast to PMMA spheres (average size of 430 nm), the enlarged lateral dimension of PMMA@Ti_3_C_2_T_*x*_ spheres (average size of 450 nm) confirms the even packaging of MXene flakes. The hollow Ti_3_C_2_T_*x*_ foam is further created after pyrolysis of PMMA@Ti_3_C_2_T_*x*_ spheres to remove the PMMA templates at 450 °C under an N_2_ atmosphere (Fig. 2d, e). The resultant Ti_3_C_2_T_*x*_ foam exhibits an interlocked and self-supporting hollow sphere structure, which can effectually avoid the self-stacking of the MXene nanosheets and provide excellent mechanical strength for improving the electromechanical properties. Meanwhile, the lamellar Cu_3_(HHTP)_2_ particles are synthesized by the solvothermal method, and their average size is determined to be 37 nm (Figs. 2f, g and S3). The high-resolution transmission electron microscopy (HRTEM) of the Cu_3_(HHTP)_2_ with a (100) crystal plane is given in Fig. S4, proving its high crystallinity. Benefited from the strong electrostatic interaction, the Cu_3_(HHTP)_2_ particles (as denoted by the yellow dashed box) are randomly and compactly grasped by the interlinked Ti_3_C_2_T_*x*_ foam to generate the Ti_3_C_2_T_*x*_@Cu_3_(HHTP)_2_ composite (Fig. 2h, i) [23]. In the HRTEM image of the Ti_3_C_2_T_*x*_@Cu_3_(HHTP)_2_ composite, a remarkable lattice fringe with the spacing of 0.2 nm of the (002) plane in Ti_3_C_2_T_*x*_ is clearly observed (Fig. 2j) [24]. This indicates the hollow Ti_3_C_2_T_*x*_ foam still remains highly crystalline even after the high-temperature annealing process. Moreover, a clear crystal lattice fringe of Cu_3_(HHTP)_2_ particles assigned to (100) plane in HRTEM of Ti_3_C_2_T_*x*_@Cu_3_(HHTP)_2_ composite is also observed [25], suggesting the coexistence of Ti_3_C_2_T_*x*_ and Cu_3_(HHTP)_2_. Finally, the uniformly spatial distribution of the Ti, Cu, C, O, and F elements of the Ti_3_C_2_T_*x*_@Cu_3_(HHTP)_2_ composite are revealed by the energy-dispersive spectroscopy (EDS) elemental mapping (Fig. 2k). In all, the above results prove the successful fabrication of the Ti_3_C_2_T_*x*_@Cu_3_(HHTP)_2_ composite. Analogous to the epidermal architecture of the skin, the close interlocking between the granular layer (spheroid Ti_3_C_2_T_*x*_) and the spine layer (Cu_3_(HHTP)_2_ particles) can augment the contact area for efficaciously capturing external stimuli and accelerate the signal transduction [12].

**Fig. 3:**
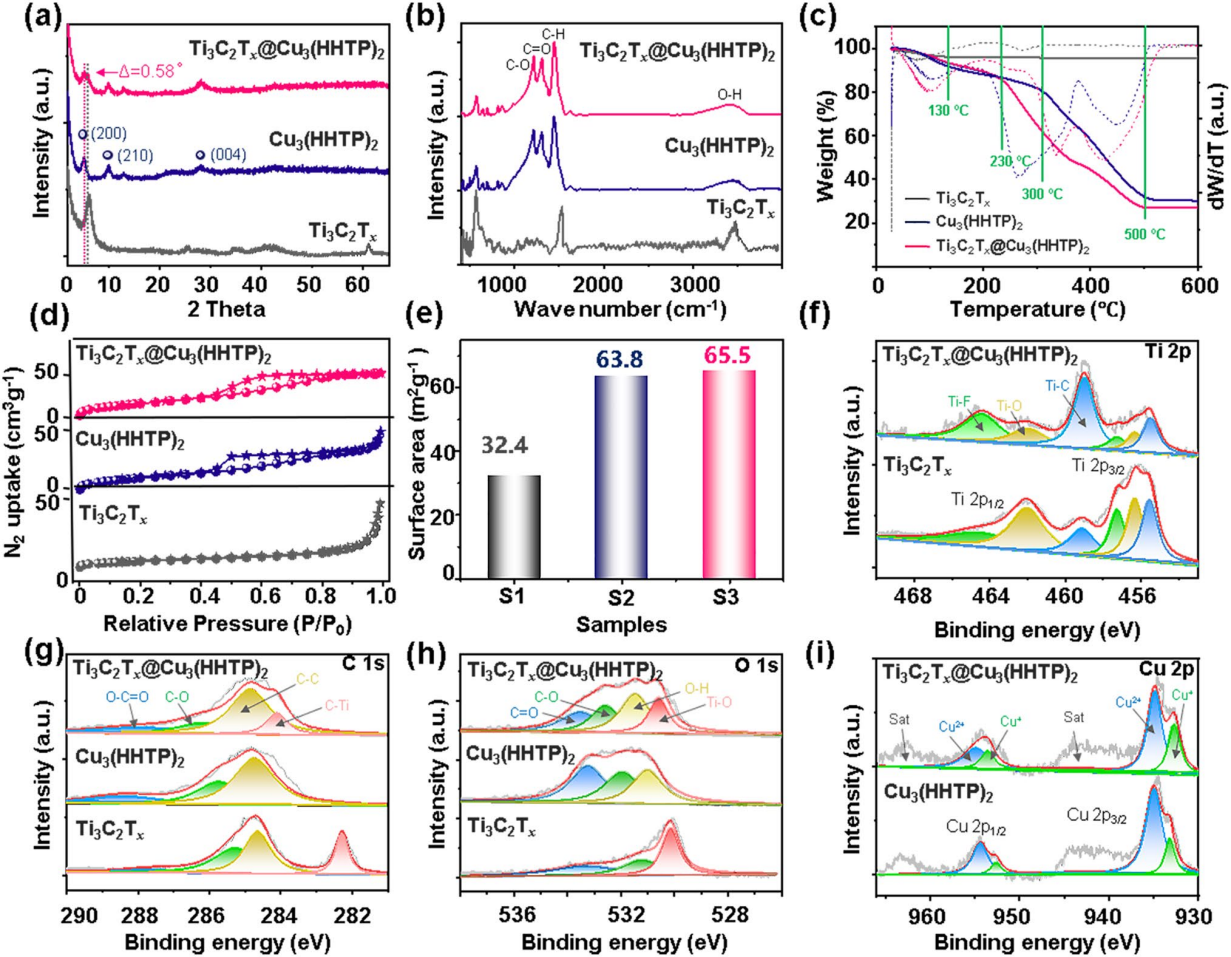
**a** XRD patterns, **b** FTIR, and **c** TGA profiles of the Ti_3_C_2_T_*x*_ foam, Cu_3_(HHTP)_2_ particles and Ti_3_C_2_T_*x*_@Cu_3_(HHTP)_2_ composite; **d** N_2_ adsorption–desorption isotherms and **e** BET surface area the Ti_3_C_2_T_*x*_ foam (S1), Cu_3_(HHTP)_2_ particles (S2) and Ti_3_C_2_T_*x*_@Cu_3_(HHTP)_2_ composite (S3); and the XPS profile of **f** Ti 2*p*, **g** C 1*s*, **h** O 1*s*, **i** Cu 2*p* orbits in Ti_3_C_2_T_*x*_ foam, Cu_3_(HHTP)_2_ particles and Ti_3_C_2_T_*x*_@Cu_3_(HHTP)_2_ composite

The X-ray diffractometry (XRD) analysis was conducted to investigate the crystal structures of the pristine Ti_3_C_2_T_*x*_ foam, Cu_3_(HHTP)_2_ particles, and Ti_3_C_2_T_*x*_@Cu_3_(HHTP)_2_ composite (Fig. 3a). Similar to the results in HRTEM images, the Ti_3_C_2_T_*x*_ foam exhibits distinctive diffraction peaks of (002) crystal planes at 2θ = 6.1° [26], confirming the robust structure even after high-temperature treatment. In addition, Cu_3_(HHTP)_2_ particles exhibit characteristic (200), (210), and (004) diffraction peaks [27], further indicating its high crystallinity. In Ti_3_C_2_T_*x*_@Cu_3_(HHTP)_2_ composite, the characteristic diffraction peaks both in Ti_3_C_2_T_*x*_ and Cu_3_(HHTP)_2_ are coexistent, showing that the self-assembly of conductive MOF particles did not disrupt the inherent structural integrity of the Ti_3_C_2_T_*x*_ framework. Moreover, the (002) diffraction peak of Ti_3_C_2_T_*x*_ in Ti_3_C_2_T_*x*_@Cu_3_(HHTP)_2_ composite downshifts slightly to 2θ = 5.5°, which is associated with the lattice expansion of the d-spacing in MXene after the introduction of MOF particles [28]. Fourier-transform infrared spectroscopy (FTIR, Fig. 3b) is further employed to analyze the structural evolution. For the Ti_3_C_2_T_*x*_ foam, the peak at 3443 cm^−1^ is mainly assigned to the O–H stretching patterns of the surface terminal functional groups. The rich –OH functional groups on the surface of Ti_3_C_2_T_*x*_ could facilitate electrostatic assembly with Cu ions of Cu_3_(HHTP)_2_ [29]. The Cu_3_(HHTP)_2_ particles exhibit three sharp vibration peaks at 1215 (C–O), 1305 (C=O), and 1445 cm^−1^ (C–H), respectively [30]. These additional peaks are retained in Ti_3_C_2_T_*x*_@Cu_3_(HHTP)_2_ composite, corroborating that the Cu_3_(HHTP)_2_ particles can be electrostatically cross-linked into the Ti_3_C_2_T_*x*_ framework.

The interlinked spherical structure bioinspired by skin contributes to enhancing the mechanical strength and structural stability of bionics materials. To investigate the thermal stability behavior of as-prepared samples, thermogravimetry analysis (TGA) and differential thermogravimetric analysis (DTG) are recorded in Fig. 3c. In the pristine Ti_3_C_2_T_*x*_ foam, a gradual weight loss starting at 100 °C is attributed to the wipe of physically adsorbed water and gases, i.e., O_2_. The subsequent slight weight loss (4.64 wt%) between 100 and 600 °C is related to the removal of the chemically adsorbed –OH groups on the Ti_3_C_2_T_*x*_ surface. This also demonstrates the abundant –OH groups on the surface of prepared MXene, which facilitates the electrostatic hetero-assembly of Ti_3_C_2_T_*x*_ and Cu_3_(HHTP)_2_ without lattice-mismatching [27]. For the Cu_3_(HHTP)_2_ particles and Ti_3_C_2_T_*x*_@Cu_3_(HHTP)_2_ composite, they exhibit similar weight loss curves trend of pyrolysis. The first attenuation stage before 130 °C associated with the evaporation of physically adsorbed water and O_2_. A slight curve descending step appears in the temperature ranging 130 ~ 300 °C for the Cu_3_(HHTP)_2_ particles and 130 ~ 230 °C for the Ti_3_C_2_T_*x*_@Cu_3_(HHTP)_2_ composite related to the vaporization of chemically adsorbed water. The last stage of weight loss is attributed to the decomposition and collapse of the organic linker (H_2_HHTP) in the Cu_3_(HHTP)_2_ frameworks, which is in keeping with the previous reports. The TGA analysis indicates that the Ti_3_C_2_T_*x*_@Cu_3_(HHTP)_2_ composite structure remains thermal stable before 230 °C, which can robustly afford the subsequent gas and pressure sensing tests at room temperature (RT). The N_2_ adsorption and desorption isotherms at 77 K were carried out to investigate the specific porous structure of the Ti_3_C_2_T_*x*_@Cu_3_(HHTP)_2_ composite (Fig. 3d). The N_2_ sorption isotherm of all three samples sharply rises at low relative pressure (type IV isotherms). In particular, the microporous structure of Cu_3_(HHTP)_2_ particles is well preserved after combination with the MOF particles. The specific surface areas determined by Brunauer–Emmett–Teller (BET) method are 32.4 m^2^ g^−1^ for pristine Ti_3_C_2_T_*x*_ foam, 63.8 m^2^ g^−1^ for Cu_3_(HHTP)_2_ particles, and 65.5 m^2^ g^−1^ for Ti_3_C_2_T_*x*_@Cu_3_(HHTP)_2_ composite, respectively (Fig. 3e). The increased specific surface area in Ti_3_C_2_T_*x*_@Cu_3_(HHTP)_2_ composite is attributed to the hollow spherical structure of Ti_3_C_2_T_*x*_ foam, which is beneficial to building more effective gas/electron transportation pathways for improving gas sensing performance. Hence, the combination of Cu_3_(HHTP)_2_ particles and Ti_3_C_2_T_*x*_ foam can effectively facilitate available accessibility of active gas species and also further accelerate the electron transmission.

The chemical composition and valence of the pristine Ti_3_C_2_T_*x*_ foam, Cu_3_(HHTP)_2_ particles, and Ti_3_C_2_T_*x*_@Cu_3_(HHTP)_2_ composite were characterized by X-ray photoelectron spectroscopy (XPS) analysis. As displayed in Ti 2*p* spectra (Fig. 3f), the presence of Ti–C (459.1/455.6 eV) peaks in the Ti_3_C_2_T_*x*_ foam reveals that a few layers of Ti_3_C_2_T_*x*_ are successfully synthesized. Additionally, the existence of Ti–F (464.5/457.3 eV) and Ti–O (462.0/456.3 eV) peaks certifies that the surface of as-prepared Ti_3_C_2_T_*x*_ foam enriches with numerous terminal functional groups, i.e., –OH, –F and –O (the calculated contents are listed in Table S1) [31]. The various functional group sites can also be observed in O 1*s* and F 1*s* of XPS curves (Figs. 3h and S5). Owing to the formation of Ti–F–Cu and Ti–O–Cu components after the assembly of Cu_3_(HHTP)_2_ particles, partial active sites of the surface organofunctional groups are occupied [32]. It can be further confirmed by a significant decrease in the peak area proportions of Ti–F and Ti–O peaks in the Ti_3_C_2_T_*x*_@Cu_3_(HHTP)_2_ composite, as listed in Table S1. In addition, the remarkable difference of C 1*s* and O 1*s* XPS peaks further provides favorable evidence of interactive grafting between Cu_3_(HHTP)_2_ and Ti_3_C_2_T_*x*_ foam. There are only three C 1*s* deconvolution peaks at 288.5, 285.7, and 284.7 eV in the Cu_3_(HHTP)_2_ particles, assigning to the O–C=O, C–O, and C–C bonds, respectively (Fig. 3g). One additional C 1*s* peak appears in the Ti_3_C_2_T_*x*_@Cu_3_(HHTP)_2_ composite, which is attributed to C–Ti (284.1 eV) of the Ti_3_C_2_T_*x*_ foam, demonstrating successful recombination of the Ti_3_C_2_T_*x*_ foam and the Cu_3_(HHTP)_2_ particles. A similar XPS peaks coupling phenomenon can be observed in the O 1*s* and Cu 2*p* spectra in Fig. 3h, i as well. In addition, as compared to the Ti_3_C_2_T_*x*_ foam, the binding energy of Ti 2*p*, C 1*s*, O 1*s*, and F 1*s* peaks in Ti_3_C_2_T_*x*_@Cu_3_(HHTP)_2_ composite all shift to the lower binding energy side, which is ascribed to the increased electron density after introducing the Cu_3_(HHTP)_2_ particles onto the Ti_3_C_2_T_*x*_ foam surface [32]. Combining the above results, it can be confirmed that the Cu_3_(HHTP)_2_ particles are compactly grafted to the Ti_3_C_2_T_*x*_ surface via the Ti–F–Cu and Ti–O–Cu chemical bridges, resulting in an excellent heterogeneous attachment for improving electronic migration.

### NO_2_ Gas Sensing Performance of Bioinspired Flexible Ti_3_C_2_T_***x***_@Cu_3_(HHTP)_2_ Sensor

**Fig. 4:**
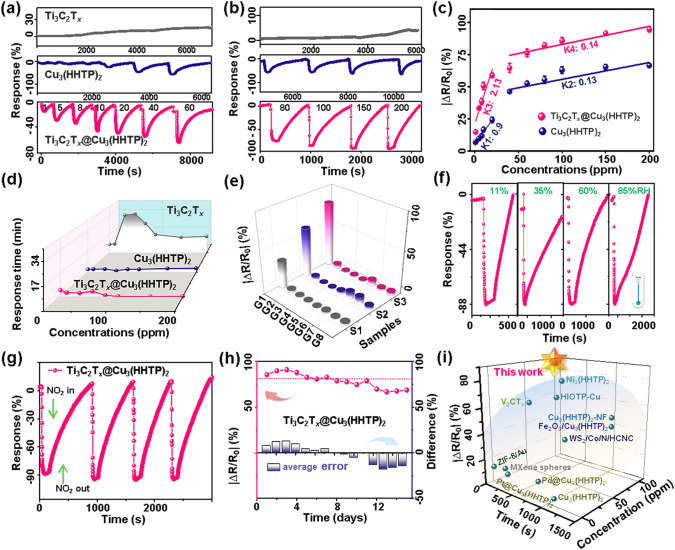
The dynamic response of various sensors to low (a, 1–60 ppm) and high (b, 80–200 ppm) concentration ranges of nitrogen dioxide (NO_2_) gas at RT, including the Ti_3_C_2_T_*x*_, Cu_3_(HHTP)_2_, and Ti_3_C_2_T_*x*_@Cu_3_(HHTP)_2_ sensors, respectively; **c** The relationship of the Cu_3_(HHTP)_2_ and Ti_3_C_2_T_*x*_@Cu_3_(HHTP)_2_ sensors to 1–200 ppm of the NO_2_ gas; **d** The response time and **e** the selectivity of three sensors to 100 ppm of various VOCs, S1-S3 represent Ti_3_C_2_T_*x*_, Cu_3_(HHTP)_2_, and Ti_3_C_2_T_*x*_@Cu_3_(HHTP)_2_ sensors, respectively; G1-G8 is NO_2_, NH_3_, H_2_S, CH_3_OCH_3_, C_6_H_7_, HCHO, CH_3_OH, C_2_H_5_OH gas, respectively; **f** Dynamic response tests of the Ti_3_C_2_T_*x*_@Cu_3_(HHTP)_2_ sensors to 100 ppm NO_2_ gas under various RHs from 11% to 85%; **g** Representative repeatable sensing tests and **h** long-term stability tests of the Ti_3_C_2_T_*x*_@Cu_3_(HHTP)_2_ sensor to 100 ppm NO_2_ gas; and **i** Response versus NO_2_ concentration and response time of the as-known state-of-the-art NO_2_-based chemiresistors

Learning from powerful olfactory perception capacity, establishing an artificial electronic nose integrated with high-performance biomimetic sensing materials can permit new possibilities in practical applications [2]. Herein, the flexible gas sensor is prepared by utilizing skin-like biomimetic Ti_3_C_2_T_*x*_@Cu_3_(HHTP)_2_ composite, which is bioinspired by the skin epidermal structure and expected to enhance the transduction of sensing signals to trace amounts of the NO_2_ stimulus. First, the Ti_3_C_2_T_*x*_@Cu_3_(HHTP)_2_ sensing material was uniformly dripped onto a flexible PI substrate with a pair of gold electrodes (Fig. S6). Figure 4a, b shows the dynamic response curves of the Ti_3_C_2_T_*x*_, Cu_3_(HHTP), and Ti_3_C_2_T_*x*_@Cu_3_(HHTP)_2_ sensors upon exposure to NO_2_ gas over low (1–60 ppm) and high (80–200 ppm) concentration ranges at RT. The resistance of pristine Ti_3_C_2_T_*x*_ sensor increases monotonously in small amplitude upon exposing NO_2_ gases (*|∆R/R*_*0*_*|*× 100% = 36% to 100 ppm), revealing the intrinsic metallic conductivity of Ti_3_C_2_T_*x*_ MXene and stronger gas adsorption of surface functional groups [33]. Due to the adsorption of strong oxidizing NO_2_ gas molecules, the electron transport is suppressed, resulting in an increased resistance of the pristine Ti_3_C_2_T_*x*_ sensor. However, an unrecoverable dynamic response appears, which is related to the irreversible gas chemisorption between NO_2_ and the surface groups of MXene in the pristine Ti_3_C_2_T_*x*_ sensor. In contrast, the Cu_3_(HHTP)_2_ and Ti_3_C_2_T_*x*_@Cu_3_(HHTP)_2_ sensors display similar negatively changed and reversible response performances after injecting NO_2_ gases. This demonstrates that the sensing behavior of the Ti_3_C_2_T_*x*_@Cu_3_(HHTP)_2_ sensor to NO_2_ gas should be mainly dominated by the Cu_3_(HHTP)_2_ particles. Due to the rational composite design, the Ti_3_C_2_T_*x*_@Cu_3_(HHTP)_2_ sensor to NO_2_ gas is significantly enhanced (*|∆R/R*_*0*_*|*× 100% = 86%), which is also higher than that of the pristine Cu_3_(HHTP)_2_ sensor (*|∆R/R*_*0*_*|*× 100% = 65%). The response of the Ti_3_C_2_T_*x*_@Cu_3_(HHTP)_2_ sensor is further optimized by modulating various mass ratios of Ti_3_C_2_T_*x*_ and Cu_3_(HHTP)_2_ particles. Figure S7 shows the dynamic response tests of three Ti_3_C_2_T_*x*_@Cu_3_(HHTP)_2_ sensors to NO_2_ gases in the range of 1–20 ppm with mass ratios changing from 1/10 to 1/40, respectively. In comparison, the Ti_3_C_2_T_*x*_@Cu_3_(HHTP)_2_ sensor with a mass ratio of 1/20 has the maximum response value to NO_2_ gas. The higher response of the Ti_3_C_2_T_*x*_@Cu_3_(HHTP)_2_–1/20 than that of Ti_3_C_2_T_*x*_@Cu_3_(HHTP)_2_–1/10 can be attributed to more interactions between the gas and the reaction sites of MOFs with higher mass ratios [34]. However, as the loading concentration of Cu_3_(HHTP)_2_ excessively increases, the agglomerated MOF particles assembled onto the hollow Ti_3_C_2_T_*x*_ spheres lead to the Ti_3_C_2_T_*x*_@Cu_3_(HHTP)_2_–1/40 sensor which exhibit a reduced response [27]. Therefore, the following sensing performance tests mainly concentrate on the Ti_3_C_2_T_*x*_@Cu_3_(HHTP)_2_ sensor with a mass ratio of 1/20, and it is further labeled as the Ti_3_C_2_T_*x*_@Cu_3_(HHTP)_2_ sensor for clarity.

The correlations between NO_2_ concentrations (1–200 ppm) and the response values of the Ti_3_C_2_T_*x*_, Cu_3_(HHTP), and Ti_3_C_2_T_*x*_@Cu_3_(HHTP)_2_ sensors are further demonstrated in Figs. 4c and S8. A higher sensitivity (defined as the slope of the calibration curve) is obtained in both Cu_3_(HHTP)_2_ (0.9% ppm^–1^) and Ti_3_C_2_T_*x*_@Cu_3_(HHTP)_2_ sensors (2.13% ppm^–1^) in the low gas concentration range (1–40 ppm), as compared to that of Ti_3_C_2_T_*x*_ sensor (0.3% ppm^–1^). Sufficient active sites in Cu_3_(HHTP)_2_ and Ti_3_C_2_T_*x*_@Cu_3_(HHTP)_2_ sensor can afford complete gas adsorption and desorption in the range of lower NO_2_ concentration, thus causing a significant resistance modulation and accelerated response/recovery kinetics. However, all the sensors tend to saturate toward high gas concentration ranges (60–200 ppm), demonstrating a relatively decreased sensitivity. This is mainly ascribed to the inherently limited sites of gas adsorption in two-dimensional materials. Noticeably, the sensitivity of the Ti_3_C_2_T_*x*_@Cu_3_(HHTP)_2_ sensor in both low and high NO_2_ concentration ranges is higher than those of the pristine Cu_3_(HHTP)_2_ and Ti_3_C_2_T_*x*_ sensor, as marked in Figs. 4c and S8. Besides, according to the dynamic response curves (Fig. 4a, b), the response/recovery times of three sensors to 10–200 ppm of NO_2_ gas are calculated, respectively (Figs. 4d and S9a). The response time of the Ti_3_C_2_T_*x*_@Cu_3_(HHTP)_2_ sensor to 100 ppm of NO_2_ gas (7 s) is 139.7 and 19.3 times faster than those of Ti_3_C_2_T_*x*_ (978 s) and Cu_3_(HHTP)_2_ (135 s) sensors. Meanwhile, the recovery times of the Ti_3_C_2_T_*x*_@Cu_3_(HHTP)_2_ sensor are comparable to the Cu_3_(HHTP)_2_ sensor, even with a much higher response value. Note that the Ti_3_C_2_T_*x*_ sensor showed an unrecoverable behavior. Such rapid dynamic process of the Ti_3_C_2_T_*x*_@Cu_3_(HHTP)_2_ sensor is ascribed to the incorporation of conductive Cu_3_(HHTP)_2_ with Ti_3_C_2_T_*x*_ foam, which significantly accelerates the carrier’s transmission by establishing extensive interconnected conductive paths [35]. In particular, the hierarchical assembly of Cu_3_(HHTP)_2_ particles and Ti_3_C_2_T_*x*_ foam can promote the effective accessibility of NO_2_ gas, which effectually shortens the transport and diffusion pathways of electrons, thus achieving the fastest response dynamics. In order to further prove the effect of hollow spherical Ti_3_C_2_T_*x*_ structure on gas-sensitive performance, we additionally prepared the *L*-Ti_3_C_2_T_*x*_@Cu_3_(HHTP)_2_ composite, which only with lamellar structure of Ti_3_C_2_T_*x*_ (*L*-Ti_3_C_2_T_*x*_). As shown in Figs. S9 and 4d, the response of *L*-Ti_3_C_2_T_*x*_@Cu_3_(HHTP)_2_ sensor to 10–40 ppm of NO_2_ gas is almost identical and the response and recover times increase 10% and 50%, as compared to that of Ti_3_C_2_T_*x*_@Cu_3_(HHTP)_2_ sensor, respectively. The change of the spherical structure of Ti_3_C_2_T_*x*_ has little effect on the gas sensitivity, which mainly plays an auxiliary role in accelerating NO_2_ gas response and recovery kinetics of the sensor. Thus, the sensitivity of the sample to NO_2_ is largely attributed to the combination of hollow spherical Ti_3_C_2_T_*x*_ foam and Cu_3_(HHTP)_2_ particles.

Moreover, the selectivity of the Ti_3_C_2_T_*x*_, Cu_3_(HHTP), and Ti_3_C_2_T_*x*_@Cu_3_(HHTP)_2_ sensors is examined as well for practical consideration. As shown in Fig. 4e, the Cu_3_(HHTP)_2_ and Ti_3_C_2_T_*x*_@Cu_3_(HHTP)_2_ sensors are sensitive to the NO_2_ gas because of inherently strong bonding ability between Cu_3_(HHTP)_2_ and NO_2_, which can be verified by the following simulated results. However, they exhibit negligible variations in resistance upon exposure to ammonia (NH_3_), hydrogen sulfide (H_2_S), acetone (CH_3_COCH_3_), toluene (C_6_H_7_), formaldehyde (HCHO), methanol (CH_3_OH), and ethanol (C_2_H_5_OH). Particularly, the Ti_3_C_2_T_*x*_@Cu_3_(HHTP)_2_ sensor exhibits a higher response to NO_2_ gas in comparison with the interfering gases (more than 12.3-fold of selectivity coefficient, Fig. S10) and other two sensors (a 1.3-fold enhancement), demonstrating the excellent selectivity of Ti_3_C_2_T_*x*_@Cu_3_(HHTP)_2_ sensor. The superior selectivity can guarantee the specific detection of NO_2_ in complex environments. The anti-humidity interference feature of the sensor is also an important indicator for the sensor in the actual detection. Figure 4f depicts the response of the Ti_3_C_2_T_*x*_@Cu_3_(HHTP)_2_ sensor toward 100 ppm NO_2_ gas under 11%–85% relative humidity (RH). As the humidity increases from 11% to 85%, the response decreases slightly from 87.7% (11% RH) to 81.9% (85% RH), indicating forceful resistance to humidity interference. The strong anti-humidity property is related to the lotus effect in the hollow spherical Ti_3_C_2_T_*x*_ surface, as further confirmed by the large water contact angle of 100 ± 0.9° (Fig. S11). The strong moisture resistance capacity endows the Ti_3_C_2_T_*x*_@Cu_3_(HHTP)_2_ sensor with the ability to discriminate trace in NO_2_ gas harsh and high-humidity environments.

In addition, the repeatability and long-term stability of the Ti_3_C_2_T_*x*_@Cu_3_(HHTP)_2_ sensor are also investigated. The hetero-assembly of Cu_3_(HHTP)_2_ can alleviate the irreversible recovery drawback of the Ti_3_C_2_T_*x*_ sensor. As displayed in Fig. 4g, the sensor’s resistance recovers to its baseline after 4 cyclic sensing tests. Moreover, the Ti_3_C_2_T_*x*_@Cu_3_(HHTP)_2_ sensor can sustain 81.3% of the original response after being stored in the 35% RH atmosphere for 14 days, which exhibits small fluctuations (< 20%) from the average response (Fig. 4h). The gas sensors’ response shows minimal variation under different bending angles. The response value bent at 60° (|∆R/R_0_|× 100% = 30.5%) can sustain 94.4% of the original response value (|∆R/R_0_|× 100% = 28.8%; Fig. S12a). In addition, the Ti_3_C_2_T_*x*_@Cu_3_(HHTP)_2_ sensor’s response remains nearly unchanged (only a 1.1% reduction) even after 10 bends at 40°, indicating strong stability and bending-resistance capacity (Fig. S12b). The outstanding long-term stability and bending-resistance benefit from the strong hollow spherical structure stability and good anti-humidity capacity of the Ti_3_C_2_T_*x*_@Cu_3_(HHTP)_2_ composite, as verified in the above TGA analysis (Fig. 3c) and humidity sensing test (Figs. 4f and S11). Besides, the assembly of Cu_3_(HHTP)_2_ particles on the surface of Ti_3_C_2_T_*x*_ foam can also effectively alleviate the local oxidation of active MXene terminals. We provide a comparison of comprehensive sensing parameters (i.e., working temperature, response, response and response time as well as RH) between recently reported MXene and MOF-based NO_2_ gas sensors and our Ti_3_C_2_T_*x*_@Cu_3_(HHTP)_2_ sensor (Fig. 4i and Table S2) [23, 36–41]. As listed, benefiting from the bionic spheroidal structure and accelerated electron transfer related to the strong interaction, the response of the Ti_3_C_2_T_*x*_@Cu_3_(HHTP)_2_ sensor is remarkably higher than that of the reported sensors with the fastest response time.

### NO_2_ Sensing Mechanism of the Bioinspired Ti_3_C_2_T_***x***_@Cu_3_(HHTP)_2_ Sensor

**Fig. 5:**
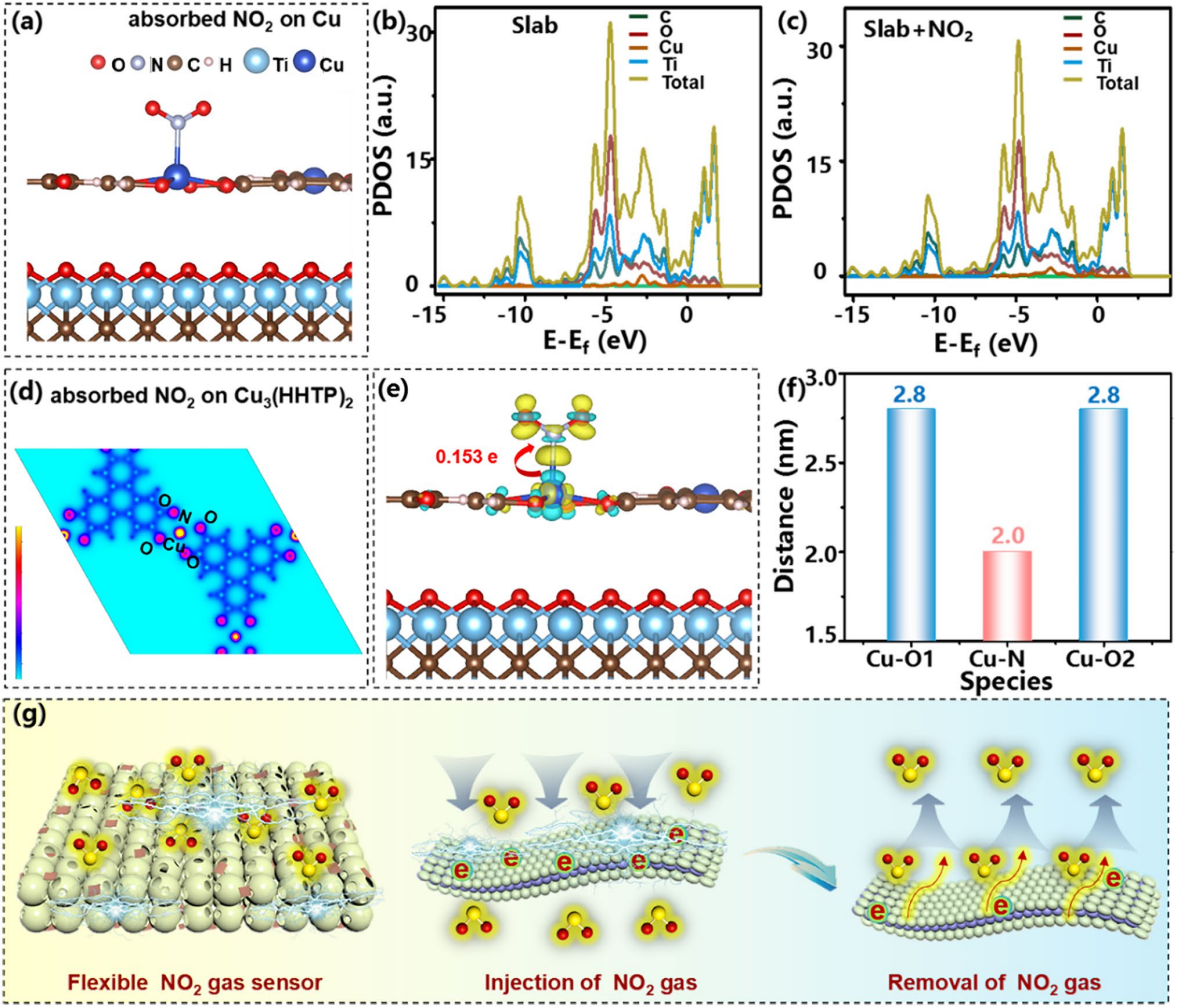
**a** Cu adsorption sites of Cu_3_(HHTP)_2_ in Ti_3_C_2_T_*x*_@Cu_3_(HHTP)_2_ composite; (b, c) PDOS of C, O, Cu, Ti atoms and total curves in Ti_3_C_2_T_*x*_@Cu_3_(HHTP)_2_ and Ti_3_C_2_T_*x*_@Cu_3_(HHTP)_2_/NO_2_ systems; **d** The charge density distribution and **e** differential charge density distribution diagram in the Ti_3_C_2_T_*x*_@Cu_3_(HHTP)_2_/NO_2_ systems; **f** The calculated distance between the Cu site and the atoms in NO_2_; and **g** Schematic diagram of the NO_2_ sensing mechanism of the Ti_3_C_2_T_*x*_@Cu_3_(HHTP)_2_ sensor

Designing a bionic interlinked spherical structure aims to both enhance the NO_2_ gas adsorption and amplify the electron signal transition, which then contributes to improving the sensor performance. To further elucidate the underlying sensing mechanism, density functional theory (DFT) calculations were executed. As proven in Figs. 4c and S8, the Cu_3_(HHTP)_2_ particles dominate the sensing response in the Ti_3_C_2_T_*x*_@Cu_3_(HHTP)_2_ sensor; it is reasonably considered as the object for in-depth theoretical analysis. Herein, three possible NO_2_ adsorption sites on Cu_3_(HHTP)_2_ particles are considered, which are Cu (Fig. 5a), C (Fig. S13a, b), and O (Fig. S13c, d) sites, respectively. In addition, two amplification regions of adsorption configuration on the top view of the C and O sites are also considered in Fig. S13e, f. Interestingly, the NO_2_ molecules tend to spontaneously adsorb onto the Cu atoms, even if NO_2_ is placed near the C and O atoms. Therefore, the most stable geometric optimization configuration model is between Cu sites of the Ti_3_C_2_T_*x*_@Cu_3_(HHTP)_2_ sensor and NO_2_ gas (Fig. 5a). The adsorption energy (*E*_*ads*_) between the Cu atom of Cu_3_(HHTP)_2_ and NO_2_ gas molecules was further calculated to be *E*_*ads*_ = − 0.174 eV as listed in Table S3, supporting the high response and selectivity of Ti_3_C_2_T_*x*_@Cu_3_(HHTP)_2_ toward NO_2_. In addition, the calculated adsorption energy of Cu active sites is negative, further indicating the spontaneity of the adsorption process between NO_2_ gas and Ti_3_C_2_T_*x*_@Cu_3_(HHTP)_2_ material. To make an in-depth understanding of the adsorption interaction, partial density of states (PDOS) curves of the C, O, Cu, and Ti atoms were plotted in Ti_3_C_2_T_*x*_@Cu_3_(HHTP)_2_ and Ti_3_C_2_T_*x*_@Cu_3_(HHTP)_2_/NO_2_ systems. As shown in Fig. 5b, c, compared to the PDOS curves of C, O, Cu, and Ti of Ti_3_C_2_T_*x*_@Cu_3_(HHTP)_2_ system, there is almost no significant change after absorption of NO_2_ gas and all curves of the Ti_3_C_2_T_*x*_@Cu_3_(HHTP)_2_/NO_2_ system does not move toward the Fermi level. This reveals that although the adsorption capacity of Cu active sites to NO_2_ is stronger, it will not destroy the stability of the structure.

As shown in Fig. 5d, the charge density distribution of Ti_3_C_2_T_*x*_@Cu_3_(HHTP)_2_/NO_2_ systems and the overlapped electron density distribution appears between Cu and N atoms. This confirms the stronger adsorption interaction between the NO_2_ molecule and Ti_3_C_2_T_*x*_@Cu_3_(HHTP)_2_ composite again. To further quantitatively analyze the adsorption affinity and charge transmission between Cu sites and NO_2_ molecules, the differential charge density distribution diagram of the Ti_3_C_2_T_*x*_@Cu_3_(HHTP)_2_ composite after absorbing the NO_2_ was executed. The electron depletion occurs in the Cu atom (denoted with blue area), while electron accumulation and charge coverage are symmetrically distributed in the N and two O atoms of NO_2_ (marked with yellow region) (Fig. 5e). This result demonstrates that the Cu sites intend to donate electrons to the surface-absorbed NO_2_ of Ti_3_C_2_T_*x*_@Cu_3_(HHTP)_2_ sensor. In addition, the corresponding distance between Cu–N (2.0 Å) atoms is closer than that of Cu–O (2.8 Å) (Fig. 5f). It is revealed that electrons prefer to flow to the N atoms instead of the O atoms in the NO_2_ molecule. The total charge number transferring from Cu sites of Ti_3_C_2_T_*x*_@Cu_3_(HHTP)_2_ to NO_2_ is determined to be 0.153 electrons, corroborating the strong carrier mobility capacity of Ti_3_C_2_T_*x*_@Cu_3_(HHTP)_2_ sensor toward NO_2_ gas.

According to the above calculation and discussion, the dynamic sensing process of the Ti_3_C_2_T_*x*_@Cu_3_(HHTP)_2_ sensor to NO_2_ gas is schematically described in Fig. 5g. Two main aspects are beneficial to improving gas sensing performance: (i) charge transfer between Cu_3_(HHTP)_2_ and adsorbed gas molecules and (ii) high conductivity of Ti_3_C_2_T_*x*_ and increased specific surface area assisted by 3D interlinked hollow spherical Ti_3_C_2_T_*x*_ structure. Upon exposure to the NO_2_ atmosphere, Ti_3_C_2_T_*x*_@Cu_3_(HHTP)_2_ sensor can rapidly adsorb NO_2_ gas via Cu sites of Cu_3_(HHTP)_2_ particles and donate electrons from the Cu sites to NO_2_ molecules. This process results in an increase in hole concentration (*p*-type sensing behavior) and a resultant resistance reduction. After the removal of NO_2_ gas, the trapped electrons can be fully released back to the Cu-MOF framework due to a reversible surface reaction, along with a resistance recovery of the sensor. The 3D interlinked electronic conduction pathways in the spherical Ti_3_C_2_T_*x*_ foam also effectively assist in forming electron networks to facilitate charge transport, which is superior to that of lamellar structure of Ti_3_C_2_T_*x*_, as verified by the accelerated response and recovery kinetics (Figs. 4d and S9c, d). Combining the excellent electron transferring capacity of Cu_3_(HHTP)_2_ particles and long-range interconnected electron migration pathways of the Ti_3_C_2_T_*x*_ foam skeletons, the Ti_3_C_2_T_*x*_@Cu_3_(HHTP)_2_ composite finally achieves rapid electron mobility and enhanced NO_2_ gas sensing performance synergistically.

### Pressure Sensing Performance of Bioinspired Flexible Ti_3_C_2_T_***x***_@Cu_3_(HHTP)_2_ Sensor

Benefiting from the bionic interlinked spherical structures and superior electrical transduction properties, besides the NO_2_ gas sensing ability, the Ti_3_C_2_T_*x*_@Cu_3_(HHTP)_2_ composite can also be fabricated as a flexible pressure sensor for physiological motion stimulation monitoring. Herein, a flexible pressure sensor is prepared by coating the Ti_3_C_2_T_*x*_@Cu_3_(HHTP)_2_ composite on the flexible polyimide (PI) membrane contacted with a pair of copper electrodes. Then, the as-fabricated pressure sensor is encapsulated by silica gel to ensure a benign ohmic contact and the size of the pressure sensor is controlled to be 1 × 1 cm^2^. In this pressure sensor, the interconnected hollow spheroidal structure embedded in the soft PI substrate can endure large compressive deformation distribution [42]. The chosen commercial PI flexible substrate is lightweight and can attach to the skin tightly, which is expected to contribute to the long-term durability of the pressure sensor.

**Fig. 6:**
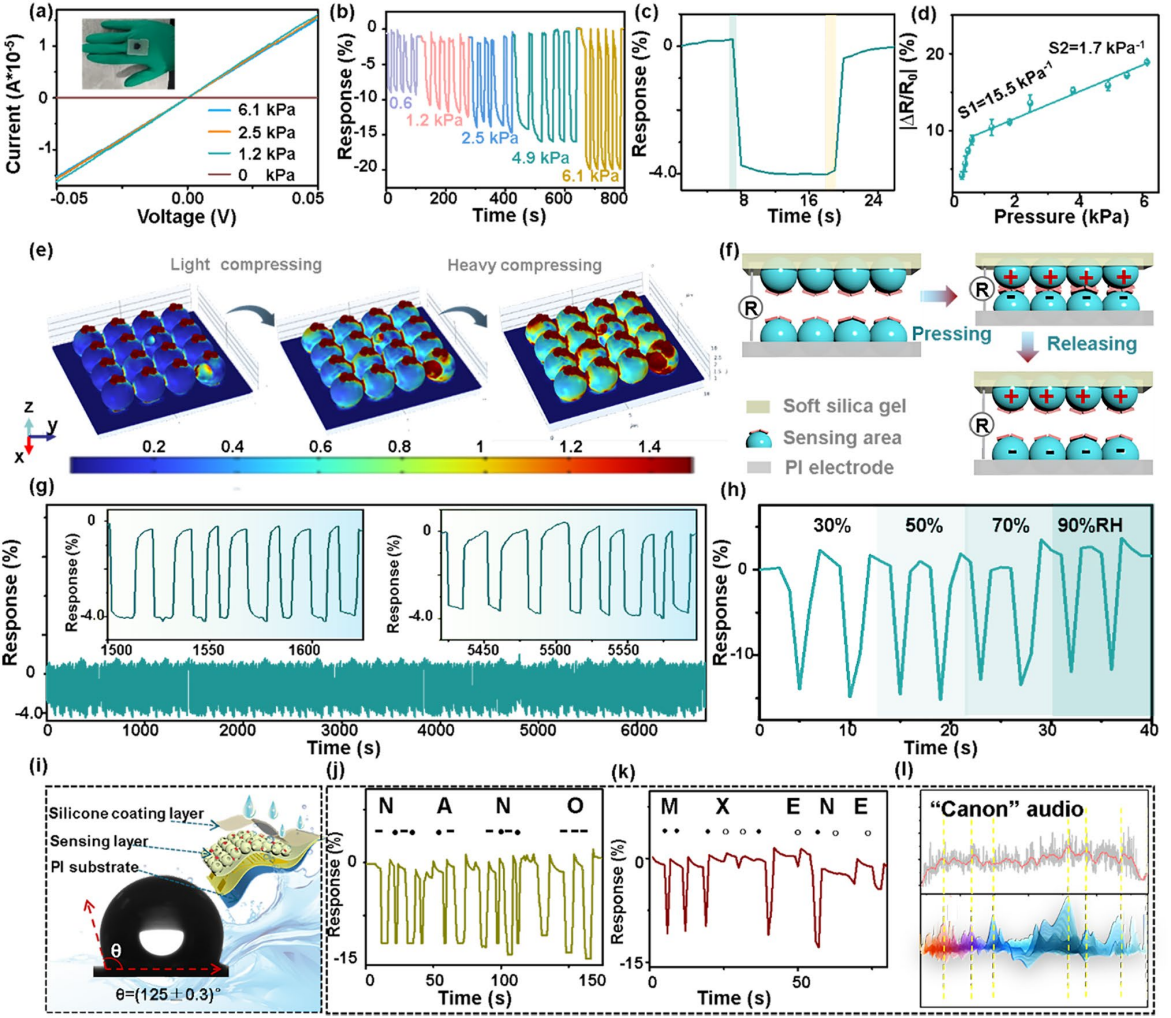
Piezoresistive performance of the Ti_3_C_2_T_*x*_@Cu_3_(HHTP)_2_ pressure sensor. **a** *I–V* curves under different pressures; **b** Resistance response to different pressure variables from 0.6 to 6.1 kPa; **c** The response/recovery times of the Ti_3_C_2_T_*x*_@Cu_3_(HHTP)_2_ pressure sensor to 0.6 kPa pressure; **d** The linear curve of the Ti_3_C_2_T_*x*_@Cu_3_(HHTP)_2_ pressure sensor in the pressure range of 0.6 to 6.1 kPa; **e** Cross-sectional view of the strain distribution simulation of the flexible Ti_3_C_2_T_*x*_@Cu_3_(HHTP)_2_ sensor under pressure; **f** Schematic diagram of pressure sensing mechanism; and **g** Repeatable response curves of the Ti_3_C_2_T_*x*_@Cu_3_(HHTP)_2_ sensor for 300 cycles of pressing/relaxing. **h** Dynamic pressure response of the sensor under various RH (from 30% to 90%); **i** Water contact angle test of the Ti_3_C_2_T_*x*_@Cu_3_(HHTP)_2_ pressure sensor (inset, the schematic diagram of the sensor structure); **j** Coding of the acronyms “NANO” via short-time and long-time heavily pressing; **k** Coding of the words “MXENE” by lightly and heavily pressing on the Ti_3_C_2_T_*x*_@Cu_3_(HHTP)_2_ sensor; and **l** The real-time response plots are monitored by a digital multimeter, as played a classic “canon” audio

Good pressure sensing properties of the sensor are conductive to discriminate the weak deformation signals generated by somatic movement. Figure 6a shows the current–voltage (*I*–*V*) curves from − 0.05 to 0.05 V of the Ti_3_C_2_T_*x*_@Cu_3_(HHTP)_2_ pressure sensor. As exhibited, the slope of the *I*–*V* curves gradually increases with stable linearity as increased applied pressure in the range of 0–6.1 kPa, well corresponding to the decreased resistance with good ohmic contact [43]. The piezoresistive variation of the Ti_3_C_2_T_*x*_@Cu_3_(HHTP)_2_ pressure sensor gradually enhances as triggered by increasing pressure from 0.6 to 6.1 kPa (Figs. 6b and S14), demonstrating the capacity of precisely capturing pressures of different levels. Moreover, the response/recovery times of the sensor are determined to be 0.9/0.9 s under 0.6 kPa pressure (Fig. 6c). The sensitivity, defined as the slope of the pressure–response calibration curve, is a key indicator for evaluating the sensing properties. As exhibited in Figs. 6d and S14, upon the relative resistance plots (ΔR/R_0_) variation versus pressure, the sensitivities of the pressure sensor present two regions: the high sensitivity S1 of 15.5 kPa^−1^ in the range from 0.3 to 0.6 kPa and the low sensitivity S2 of 1.7 kPa^−1^ ranging from 0.6 to 6.1 kPa. Such high sensitivity is attributed to increased 3D deformation of skin-bioinspired hollow spherical structure. A comparison of sensing performance between recently published pressure sensors and our Ti_3_C_2_T_*x*_@Cu_3_(HHTP)_2_ sensor is summarized in Table S4 [20, 23, 44–51]. Although the sensitivity of the as-prepared device is comparable, further improvement of pressure sensitive performance in future work will be explored.

Benefiting from interlocked microstructures bioinspired by the granular and spinous layers of the epidermis, the Ti_3_C_2_T_*x*_@Cu_3_(HHTP)_2_ pressure sensor can sensitively capture external pressure stimuli. According to previous reports, the sensing mechanism of the pressure sensors currently includes the following: (1) contact resistance effect, (2) percolation theory, (3) tunneling effect, (4) fracture and crack propagation, and (5) semiconductor piezoresistive effect [52]. Herein, the contact resistance effect plays a leading role in this work. In Ti_3_C_2_T_*x*_@Cu_3_(HHTP)_2_ pressure sensor, the total resistance is composed of electrode resistance and active layer resistance. The contact resistance of Ti_3_C_2_T_*x*_ and Cu_3_(HHTP)_2_ mainly affects the resistance of the active layer, since the electrode resistance is constant. In our work, the design concept emphasizes creating 3D interlinked hollow spherical composite structures in sensitive materials to enhance sensor sensitivity to NO_2_ and pressure. (i) The 3D interlocking Ti_3_C_2_T_*x*_ spherical structure can support a larger compression deformation, thereby generating the increased conductive area and conductive path, dominating the pressure sensing process. This can be confirmed by the decreased sensitivity of *L*-Ti_3_C_2_T_*x*_@Cu_3_(HHTP)_2_ sensor without Ti_3_C_2_T_*x*_ spherical structure (K1 = 0.8 kPa^−1^ and K2 = 0.25 kPa^−1^, Fig. S15a, b), as compared to that of Ti_3_C_2_T_*x*_@Cu_3_(HHTP)_2_ (K1 = 15.5 kPa^−1^ and K2 = 1.7 kPa^−1^, Fig. 6d) with spherical structure. (ii) The Cu_3_(HHTP)_2_ can assist spherical Ti_3_C_2_T_*x*_ to modulate the contact resistance due to its larger resistance, thus improving the overall pressure response. As compared in Fig. 6d, the pressure response of Ti_3_C_2_T_*x*_@Cu_3_(HHTP)_2_ sensor to 6.1 kPa is 19%, while that of the spherical Ti_3_C_2_T_*x*_ sensor without Cu_3_(HHTP)_2_ particles modification is only 11% (Fig. S15c).

To further elucidate the pressure-induced sensing mechanism, a simplified ball-and-panel geometric model of the Ti_3_C_2_T_*x*_@Cu_3_(HHTP)_2_ pressure sensor is established using finite element method (FEM). As shown in Figs. 6e and S16–S18, stepwise deformation (from a sphere to an ellipsoid) and increased displacement occur at the interlinked interface between the microtome and panel as pressure gradually increases. The stress concentrates at the nano-interlocked interface, indicated by the color changes from blue to cyan to red. The stress in the pressure sensor is closely dependent on the variation of the contact surface area and the conductive channel distance of the sensitive material. To clarify the pressure sensing process, the enlarged geometric model was used to reveal resistance change under applied pressure (Fig. S18). For simplification, the area (S) of the Ti_3_C_2_T_*x*_ ball is assumed to remain constant when the sphere is compressed into an ellipse, as shown in Fig. S18. Note that the length of any chord in the ellipse (x_2_) is inversely correlated with the minor axe (b). As illustrated in Figs. 6f and S18, applying pressure enlarges the contact area (A) between Ti_3_C_2_T_*x*_ and Cu_3_(HHTP)_2_ and shortens the conductive channel (d2), leading to a significant decrease in resistance. Upon pressure release, the resistance and deformation fully recover to their initial state. Therefore, the Ti_3_C_2_T_*x*_@Cu_3_(HHTP)_2_ pressure sensor is very sensitive to pressure change, even as low as 120 Pa (Fig. S19a).

In addition, a constant sensing curve of the Ti_3_C_2_T_*x*_@Cu_3_(HHTP)_2_ sensor under no pressure load is provided in Fig. S19b, indicating an excellent resistance stability. The Ti_3_C_2_T_x_@Cu_3_(HHTP)_2_ pressure sensor can endure 300 pressing/relaxation cycles (Fig. 6g), demonstrating excellent reliability. As shown in Fig. 6g, almost no fluctuations appear in the cyclic response curves during the whole sensing process. What’s important is that the repeatable response still remains robust because the fracture propagation of conductive paths can be effectively prevented by the hierarchical interlinked hollow sphere structure. This proves that the as-designed flexible pressure sensor has good repetitive durability and mechanical flexibility against long-duration dynamic pressing-relaxation testing. Moreover, the pressure response still maintains 97% of the original response even after 6 months (Fig. S20a), and the response value of the flexible Ti_3_C_2_T_*x*_@Cu_3_(HHTP)_2_ pressure sensor remains unchanged even after 20 bends, indicating excellent electromechanical stability of the sensor (Fig. S20b, c). The anti-humidity interference feature is also a key sensing parameter to meet the challenge of all-weather conditions. Figure 6h displays the dynamic piezoresistive response of the flexible Ti_3_C_2_T_*x*_@Cu_3_(HHTP)_2_ pressure sensor under a different RH ambient. There is almost no response variation as humidity increases from 30% to 90% RH ambient. According to the hydrophobic angle test, the contact angle of the pressure sensor is determined to be 125 ± 0.3°, confirming the strong waterproof capacity of the flexible sensor with the protection of a hydrophobic silicone package (Fig. 6i). Thus, the as-designed pressure sensor possesses a strong capacity to shield the attack of water molecules or ambient oxygen, which can guarantee a stable detection in sweat-prone human skin.

Particularly, the ultra-thin and soft PI substrate, as well as the waterproof-ability of the surface package, endows the as-designed pressure sensor with natural accessibility to adhere onto the human skin, thus carrying a great potential for multifunctional human-health motion monitoring. To further evaluate the capability to capture the imperceptible physical action signals, the Ti_3_C_2_T_*x*_@Cu_3_(HHTP)_2_ pressure sensor is applied to the detection of tiny human kinematic vibrations. As the experimenter controls the exerting intensity of blowing, tapping, and pressing actions, the sensor automatically exhibits different response values (Fig. S21). This indicates that the Ti_3_C_2_T_*x*_@Cu_3_(HHTP)_2_ pressure sensor has superior electromechanical sensing characteristics, offering a promising opportunity for deployable real-sensing applications to human smart-health monitoring.

Based on the above analysis, the advantages of high response, short response time, repetitive durability, hydrophobicity, and great bending-resistance of the Ti_3_C_2_T_*x*_@Cu_3_(HHTP)_2_ pressure sensor are confirmed. This entitles the pressure sensor with promising possibilities in the information record and encryption transmission. Therefore, the Ti_3_C_2_T_*x*_@Cu_3_(HHTP)_2_ pressure sensor is employed to discriminate a series of different words for information communication by delivering the English letters in the way of Morse code. There are two expressways applied in our work to define the Morse code: (i) short- and long-duration heavy pressing are defined as the dots and dashes mode and (ii) light and heavy short-duration pressing are defined as the hollow dots and solid dots, respectively (Fig. S22a). As displayed in Figs. 6j and S22b, the acronyms of “NANO,” “JLU,” and “SCI” are visualized and recorded via the as-prepared pressure sensor. In addition, the word messages of “MXENE,” “MOF,” and “SENSING” were, respectively, recorded by applying the light and heavy pressing modes (Figs. 6k and S22c). Note that the pressure sensor can immediately respond and present robust signal stability. Such high-performance finger-pressing sensing is hopefully responsible for the encrypted transmission of important information.

Furthermore, another way of propagating the signal is through the eardrum; although the oscillation generated by the air is weak, the Ti_3_C_2_T_*x*_@Cu_3_(HHTP)_2_ pressure sensor can still identify the imperceptible vibration signature [43]. For verification, the Ti_3_C_2_T_*x*_@Cu_3_(HHTP)_2_ sensor is utilized as a sound sensor to fully capture and record music vibrations. It can display the loudness, pitch, timbre of the sound, and so on, bestowing broad prospects in music melodies visualization. Thus, the flexible sensor was further mounted on a commercial speaker and its response plots are monitored by digital multimeters (Fluke, 8846A) as played a classic “canon” audio on the phone. As demonstrated in Figs. 6l and S23, the response change on the y-axis indicates the intensity of sound (volume and characteristic peak), and the time span on the x-axis represents the play time. It is clearly observed that the characteristic response peaks of the Ti_3_C_2_T_*x*_@Cu_3_(HHTP)_2_ pressure sensor match well with the music melody, and its response is fast and stable. Moreover, there is a good consistency with the original canon audio signal, which exhibits a significant difference in response amplitude between each music chorus and low registers. From the continuously changing vibration wave features and good music-reproduction ability, it is believed that the designed Ti_3_C_2_T_*x*_@Cu_3_(HHTP)_2_ pressure sensor is promising in realizing the function of sound visualization. This is conducive to simplifying the route of sound acquisition and recognition, which bestows great application prospects for automatic speech recognition.

### Wearable and Smart Health-Monitoring Application Assistant with Machine Learning Algorithm

It is reported that NO_2_ can act as exogenous stimulus to exacerbate asthma [53], and World Health Organization recommends a 1 h-guideline of 200 µg m^−3^ (100 ppb). Moreover, anomalous somatic dyskinesia, such as abnormal breathing and weakness in the limbs, are considered early warning symptoms of asthma’s disease [4, 54]. However, the traditional medical monitoring equipment makes it difficult to simultaneously recognize these hazardous gases and subtle characteristics of abnormal physiological signals. The Ti_3_C_2_T_*x*_@Cu_3_(HHTP)_2_ have a significant different response and recovery dynamic when responded to gas or pressure. However, to reliably monitor the gas or pressure stimuli, the pressure sensor was packed with silicone gel to avoid the gas interference, which has been presented in Sect. 2.2. According to the gas sensing test results, the NO_2_ response is almost unaffected by external forces (Fig. S12). Moreover, the pressure response is also almost unaffected by external NO_2_ gas and humidity under the protection of silica gel layer (Fig. S24) and (Fig. 6h). Based on the above results, the subsequent practical application is carried out. The powerful capability to accurately detect both NO_2_ gas and pressure without mutual cross-interferences bestows the possibility for multiplex signal detection of intelligent wearable systems.

**Fig. 7:**
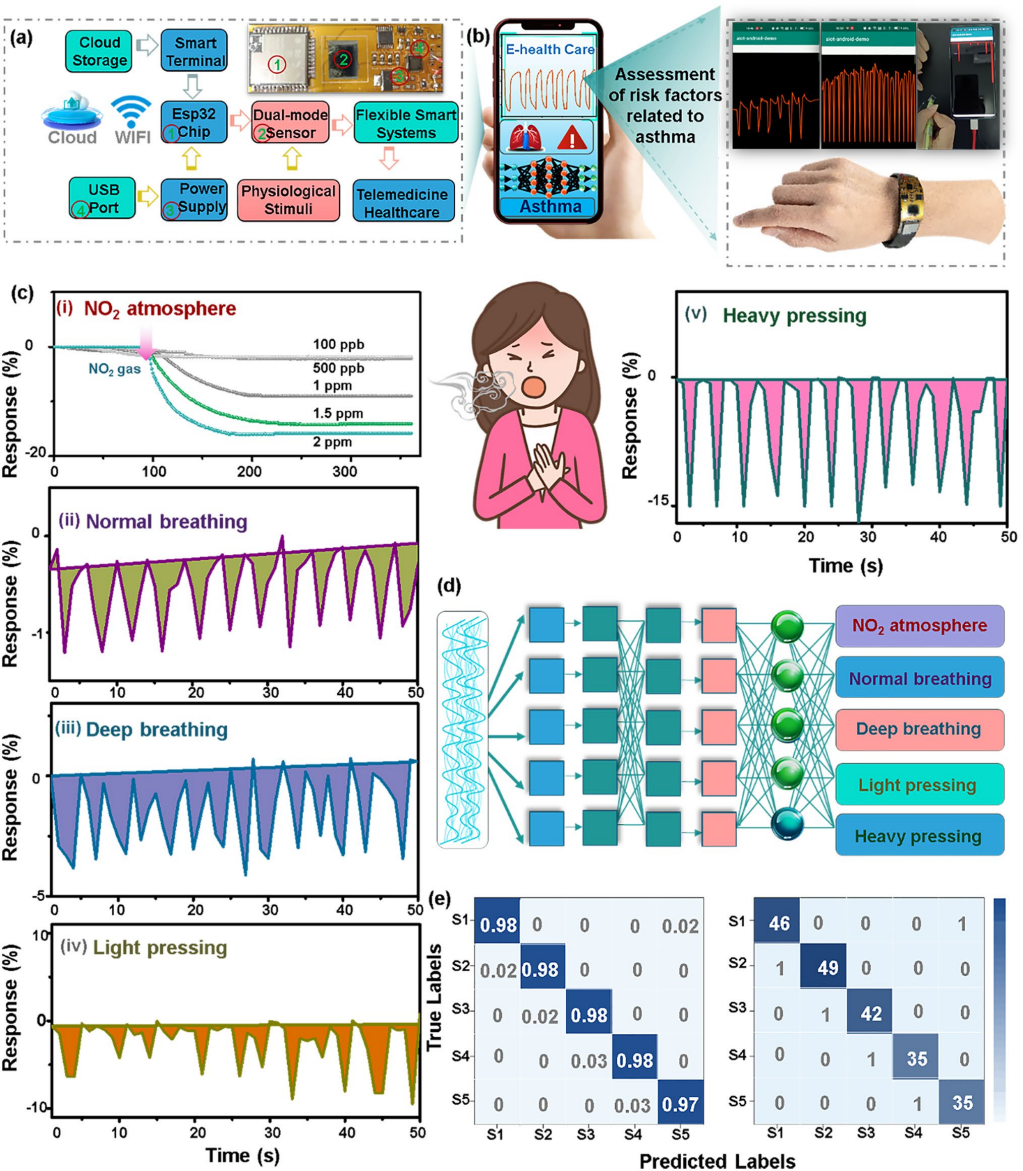
Flexible wearable alarming system for monitoring of somatomotor signals of asthma; **a** Schematic illustration of the flexible smart wearable alarming system including ESP32 chip, dual-mode sensor, power supply and wireless data transmission by WIFI, etc*.*; **b** Optical image showing that flexible dual-mode sensor is integrated into flexible printed circuits to detect different pressures; **c** The real-time response of the wearable alarming system to *i* the NO_2_ atmosphere, (*ii, iii*) normal and deep breathing, and (*iv, v*) light and heavy pressing; and **d** Schematic diagram of the basic structure of 1-D CNNs. **e** The confusion matrix of five somatomotor patterns: S1, S2, S3, S4, and S5 representing light breathing (normal patterns), deep breathing (wheezing patterns), light and heavy pressing (limb weakness patterns), and NO_2_ atmosphere to exacerbate asthma, respectively

Accordingly, a smart wearable alarming system in a wireless mode is designed by integrating the dual-mode flexible Ti_3_C_2_T_*x*_@Cu_3_(HHTP)_2_ gas and pressure sensor on a flexible printed circuit board (FPCB) with a size of 5 × 2 cm^2^ (Fig. 7a). To ensure the miniaturization design of the wearable alarming system, the resistive sensor is selected as the parameter to assess gas and pressure responses. For example, the capacitor acquisition module is normally with larger size and more complex circuit than the resistance acquisition circuit. The analyzed response profiles of NO_2_ gas and pressure can be real-time visualized via the self-developed application page in a smartphone, as the wireless WIFI module is connected to the removable smart terminal. It displays actual pictures of some representative sensing scenarios in the application, in which the flexible sensor patch adheres to the facemask and other parts of the body using the double-sided tape to detect different breathing patterns and human motions (Fig. 7b). Apart from the response signals of the NO_2_, some potential syndromes of asthma patients could synchronously be discriminated from multiple perspectives (Fig. 7c). As expected, the Ti_3_C_2_T_*x*_@Cu_3_(HHTP)_2_ sensor can respond to 100 ppb–2 ppm of NO_2_ gas immediately in a simulated NO_2_ environment that aggravates asthma attack (panel I in Figs. 7c, S25, and Movie S1). In addition, the pressure sensor utilizing the same Ti_3_C_2_T_*x*_@Cu_3_(HHTP)_2_ composite can also distinguish the difference in breathing patterns. Particularly from panel *iii* of Figs. 7c, S26, and Movie S2, it clearly exhibited an approximately threefold higher resistance transition to deep breathing stimuli than that of normal breathing (panel *ii* of Fig. 7c). As shown in the panel *iv* and *v* of Figs. 7c, S27, S28, and Movie S3, a significant response of the flexible pressure sensor closely depends on different degrees of exertion, bestowing feasible to identify limb motion related to asthma. The flexible smart sensing system exhibits lower response signals to gentle pressing and simulative dysgraphia induced by myasthenia of limbs for asthma patients, different from the higher resistance transitions occurring in healthy people. In short, the flexible smart wearable alarming system can promptly respond to five model classifications of somatic vibration disorders related to asthma sufferers.

In addition, factors like external NO_2_ gas, abnormal expiratory behavior, and finger exertion levels can be collectively assessed to gauge asthma attack likelihood (refer to panel i of Figs. 7c, e, S25 and S29). Machine learning algorithms can endow sensors the capability to “think,” thereby accurately classifying gases and physiological responses. Moreover, a machine learning algorithm based on 1-D deep convolutional neural networks (CNNs) is further conducted to validate the accuracy of the proposed sensors’ recognition of breathing and somatomotor patterns. We demonstrate the basic structure of the 1-D convolutional neural network as depicted in Fig. 7d. Additionally, a total of 2203 sets of data (each set including 16 points) for the five modes of somatomotor were collected for this testing. It can significantly speed up the convergence velocity of model iteration and reduce overfitting by normalizing the physiological signals through the functional Eq. S2. Figure 7e exhibits the plot of the confusion matrix, and the overall identification accuracy can reach up to 97.6%. The convergence results of the model are testified in Fig. S29. The model classifications of light breathing (normal patterns, S1), deep breathing (wheezing patterns, S2), light and heavy pressing (limb weakness patterns, S3 and S4), and NO_2_ atmosphere (S5, NO_2_) have attained an accuracy of close to 100%. The demonstration of recognition of five breathing patterns is provided in the supporting information. In brief, the flexible smart wearable alarming system demonstrates remarkable sensitivity, repetitive stability, and high precision to detect both NO_2_ gas and human kinematics tremors with the aid of machine learning algorithm analytics, perfectly realizing the precise differentiation of similar and weak physiological motor signals. This indicates that the as-designed wearable alarming system possesses a feasible and potential application in recording asthma patients’ various pathological movement signals from multiple perspectives.

## Conclusion

In summary, the Ti_3_C_2_T_*x*_@Cu_3_(HHTP)_2_ composite bioinspired by 3D interlocked spherical structures and encoding information functions of the skin is successfully fabricated. Notably, this design enables the simultaneous detection of NO_2_ gas and pressure while achieving non-interfering resistance output signals. In particular, superior characteristics such as the high response (*|∆R/R*_*0*_*|*× 100% = 86% to 100 ppm) to NO_2_ gas, fast response speed (7 s), 14-day long-term stability, and excellent hydrophobicity have been realized, with the behind sensing mechanism further confirmed by density function theory (DFT) calculations. Meanwhile, efficient pressure responses within a 0–6.1 kPa range, speedy response, and recovery rates (0.9/0.9 s), along with robust repeatability over 300 cycles, were also established, all affirmed further by finite element analysis. The excellent signal transduction capacities endow flexible devices with powerful functionalities in acoustic signature perception, Morse code-encrypted message communication, and physiological motion perception. Finally, by incorporating the dual-mode sensor and a WIFI transmission module, a flexible and smart warning platform was developed. The self-developed smart wearable alarming system can precisely identify abnormalities related to asthma with the aid of a machine learning algorithm. Along with recognizing the NO_2_ signals and diverse somatic motor, it results in a classification accuracy of up to 97.6%. This work provides a realistic application prospect to build a flexible, intelligent electronic wearable warning system in the realm of telemedicine diagnosis.

## Supplementary Information

Below is the link to the electronic supplementary material.

Supplementary file1 (DOCX 4416 KB)

Supplementary file2 (MP$ 3139 KB)

Supplementary file3 (MP$ 16071 KB)

Supplementary file4 (MP$ 6146 KB)
