# Supplementary material for: A Flexible Smart Healthcare Platform Conjugated with Artificial Epidermis Assembled by Three-Dimensionally Conductive MOF Network for Gas and Pressure Sensing
Source: Nanomicro Lett. 2024 Oct 25;17:50. doi: 10.1007/s40820-024-01548-5 (PMC11511809; doi:10.1007/s40820-024-01548-5)
Supplement: Supplementary file 1 — Supplementary file1 (DOCX 4416 KB) [file 40820_2024_1548_MOESM1_ESM.docx]

Supporting Information for

**A Flexible Smart Healthcare Platform Conjugated with Artificial Epidermis Assembled by Three-Dimensionally Conductive MOF Network for Gas and Pressure Sensing**

Qingqing Zhou^1^, Qihang Ding^2^, Zixun Geng^1^, Chencheng Hu^1^, Long Yang^1^, Zitong Kan^1^, Biao Dong^1^, Miae Won^2,3^, Hongwei Song^1^, Lin Xu^1,※^, Jong Seung Kim^2,3,※^

^1^ State Key Laboratory of Integrated Optoelectronics, College of Electronic Science and Engineering, Jilin University, Changchun 130012, People's Republic of China

^2^ Department of Chemistry, Korea University, Seoul 02841, Republic of Korea

^3^TheranoChem Incorporation, Seoul 02856, Republic of Korea

^※^Corresponding authors. E-mail: [linxu@jlu.edu.cn](mailto:linxu@jlu.edu.cn) (Lin Xu); [jongskim@korea.ac.kr](mailto:jongskim@korea.ac.kr) (Jong Seung Kim)

**S1 Experimental captions**

**S1.1 Chemicals**

Copper acetate (Cu(AC)_2_) and 2,3,6,7,10,11-Hexahydroxytrimethylene hydrate (HHTP) were purchased from Shanghai Aladdin Biochemical Technology Co., LTD. Methyl methacrylate (MMA, 99%) and lithium fluoride (LiF, AR) were obtained from Shanghai Macklin Biochemical Technology Co., Ltd (China). TI_3_AlC_2_ chunk was supplied by the Yiyi Technology Co., LTD. Potassium persulfate (K_2_S_2_O_8_, 99.5%) and 2,2'-Azobis(2-methylpropionitrile) (AIBN), toluene (C_7_H_8_, AR), formaldehyde (HCHO, AR), ethanol (C_2_H_5_OH, AR), methanol (CH_3_OH, AR), formaldehyde (HCHO, AR) and acetone (CH_3_COCH_3_, AR) were purchased from Beijing Chemical Plant of China. Hydrochloric acid (HCl, AR) was acquired from Tianjin Fengchuan Chemical Reagent Technology Co., LTD. Standard NO_2_, H_2_S and NH_3_ gases for the sensing measurement were obtained from Dalian Special Gases Co., Ltd (China). No further purification was performed for all chemical reagents and the deionized (DI) water was utilized in the whole experimental process.

**S1.2 Synthesis of the** **Ti_3_C_2_T_x_ MXene nanosheets**

For delamination, 2 g of LiF powder was firstly added into the 20 mL of HCl (9 M) aqueous solution and constantly stirred for 15 min at 35℃. Then, 1 g of Ti_3_AlC_2_ powder was decanted in the above solution and kept etching at 35℃ for 24 h. After the etching procedure, the collected precipitates were dispersed in DI water and further purified by repetitive centrifugation (3500 rpm for 10 mins) until the PH value of centrifuge supernatant approached to 6. To fabricate the delaminated Ti_3_C_2_T*_x_* flakes, the as-produced precipitates were redispersed into 200 mL of DI water and continuously sonicated for 1 h in the ice-water bath. Finally, after centrifugation (3500 rpm) for 30 mins, the upper supernatant is collected as the few-layered Ti_3_C_2_T*_x_* flakes, and the resultant sediment is multi-layered Ti_3_C_2_T*_x_* nanosheets.

**S1.3** **Preparation of the 3D maroporous** **Ti_3_C_2_T_x_ foam**

Firstly, the as-prepared monodispersed polymethylmethacrylate (PMMA) macro-spheres with 430 nm diameters were synthesized by emulsion polymerization according to the previous literature [[S1](#_ENREF_1)]. To facilitate the electrostatic assembly between Ti_3_C_2_T*_x_* flakes and PMMA spheres, the 40 mL of above-fabricated Ti_3_C_2_T*_x_* flakes colloid suspension (2.5 mg/mL) was added into 6 mL of PMMA aqueous solution (70 mg/mL) and sonicated for 10 mins. After that, the Ti_3_C_2_T*_x_*/PMMA hybrid film were prepared by filtering through a Celgard 3501 polypropylene membrane and then peeled off after drying for 10 h at RT. Finally, the Ti_3_C_2_T*_x_*/PMMA hybrid film were annealed at 450°C under N_2_ atmosphere for 1.5 h with a heating rate of 10°C /min to remove the PMMA spheres, yielding the hollow Ti_3_C_2_T*_x_* foam.

**S1.4 Preparation of the 3D maroporous Ti_3_C_2_T_x_@Cu_3_(HHTP)_2_ composite**

To fabricate Cu_3_(HHTP)_2_ nanosheets, 0.6 mmol of Cu(AC)_2_ and 0.5 mmol of HHTP were respectively solved into 5 mL of ethanol and then vigorously stirred at 45°C for 30 mins to form the uniform mixture solution. The resultant Cu_3_(HHTP)_2_ were collected by vacuum filtrated through the polypropylene membrane. Afterwards, macroporous hollow Ti_3_C_2_T*_x_* films and Cu_3_(HHTP)_2_ with mass ratio of 1:10, 1:20 and 1:40 were separately mixed, respectively, to optimize the gas sensing performance of the Ti_3_C_2_T_x_@Cu_3_(HHTP)_2_ composite. They were marked as Ti_3_C_2_T_x_@Cu_3_(HHTP)_2_-1/10, Ti_3_C_2_T_x_@Cu_3_(HHTP)_2_-1/20 and Ti_3_C_2_T_x_@Cu_3_(HHTP)_2_-1/40. As revealed from the sensing performance tests, the Ti_3_C_2_T_x_@Cu_3_(HHTP)_2_-1/20 composite displayed the optimal sensing behavior, it is mainly investigated and discussed, which further abbreviated as Ti_3_C_2_T_x_@Cu_3_(HHTP)_2_ composite for convenience.

**S1.5 Characterizations of various** **samples**

The morphological architecture of various samples is confirmed by field-emission scanning electron microscope (FESEM, JEOL JSM-7500F, Japan), Transmission electron microscopy (TEM) and high-resolution transmission electron microscopy (HRTEM). To determine the structure of the samples, X-ray diffractometer (Tokyo, Japan) with a monochromatised Cu target radiation source (λ= 1.5406 Å) are conducted. Fourier transform infrared spectroscopy (FTIR) spectra are recorded on a Vertex 80 V (Bruker) FTIR spectrometer in the range of 400-4000 cm^-1^. The thermal decomposition behavior of samples is analyzed by thermogravimetric analysis (TGA, Q500, TA) and differential thermogravimetry analysis (DTG). The chemical composition of samples is confirmed by X-ray photoelectron spectra (XPS, Thermo Fisher Scientific, Waltham, MA, USA).

**S1.6 Computational details**

Density functional theory (DFT) calculations were performed using the CP2K package. [[S2](#_ENREF_2)] We have chosen the DZVP-MOLOPT-SR-GTH as basis set and pseudopotential to describe the ionic cores with a cutoff energy of 400 Rydberg in all relaxation processes. The convergence criterion for the maximum force is set as 1×10^−3^ atomic units. The k-points were 1×1×1 for geometry optimization and energy calculation. The atomic structures were analyzed by using the VESTA code [[S3](#_ENREF_3)].

The adsorption energy (***E_ads_***) is calculated via the following formula (S1):

***E_ads_* = *E_total_ – E_slab_ –***$\boldsymbol{E}_{\boldsymbol{NO}_{\boldsymbol{2}}}$

where ***E_total_***, ***E_slab_*** and $\boldsymbol{E}_{\boldsymbol{NO}_{\boldsymbol{2}}}$ represent the DFT energies of adsorbates with substrates, the substrates and NO_2_ gas adsorbates, respectively. The more negative this ***E_ads_*** value is, the stronger the adsorption.

**Supplementary Figures**


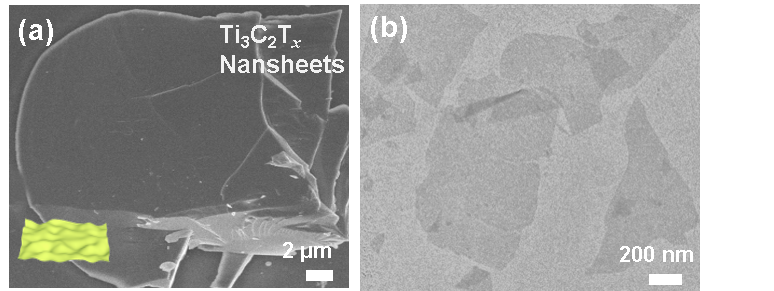


**Fig. S1** SEM and TEM images of few Ti_3_C_2_T*_x_* flakes


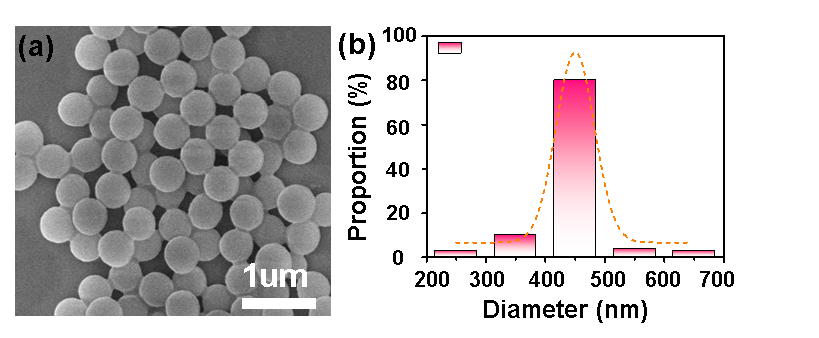


**Fig. S2** SEM image and size statistics of the PMMA spheres


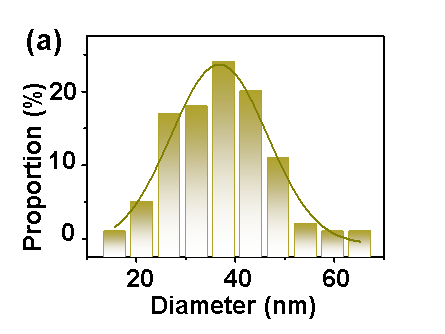


**Fig. S3** The size statistics of the Cu_3_(HHTP)_2_ particles


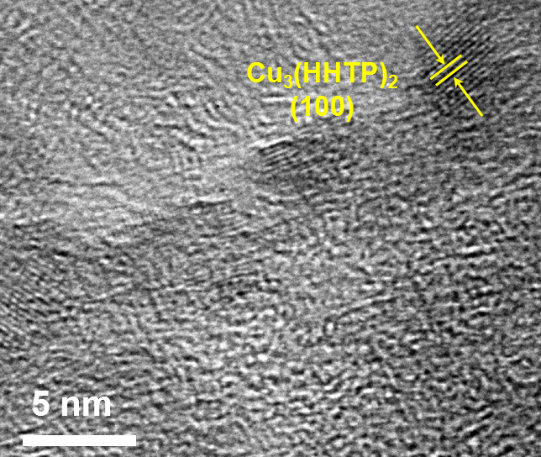


**Fig. S4** HRTEM image of the Cu_3_(HHTP)_2_ particles


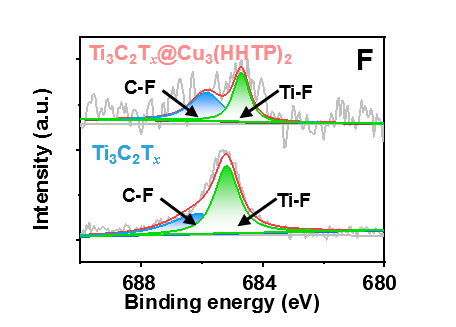


**Fig. S5** F 1s orbits in Ti_3_CT*_x_* foam and Ti_3_C_2_T*_x_*@Cu_3_(HHTP)_2_ composite


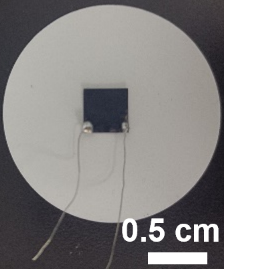


**Fig. S6** The flexible Ti_3_C_2_T*_x_*@Cu_3_(HHTP)_2_ gas sensor


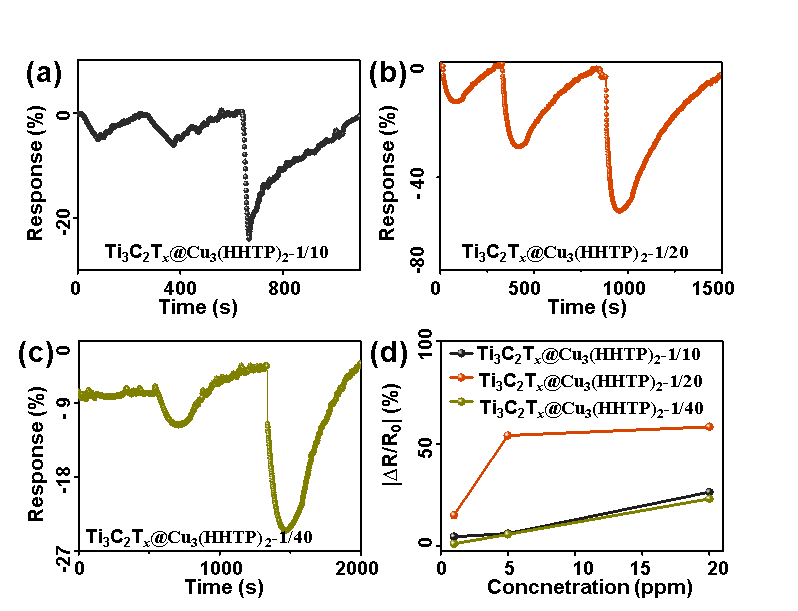


**Fig. S7** The response of the flexible Ti_3_C_2_T*_x_*@Cu_3_(HHTP)_2_ sensor to 1, 5 and 20 ppm NO_2_ gas, respectively, and the different ratios of Ti_3_C_2_T*_x_* and Cu_3_(HHTP)_2_ is 1/10, 1/20 and 1/40


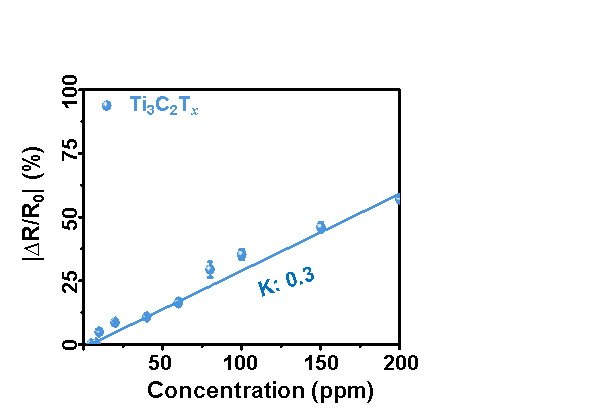


**Fig. S8** The linear relationship of the Ti_3_CT*_x_* sensors to 1-200 ppm of the NO_2_ gas


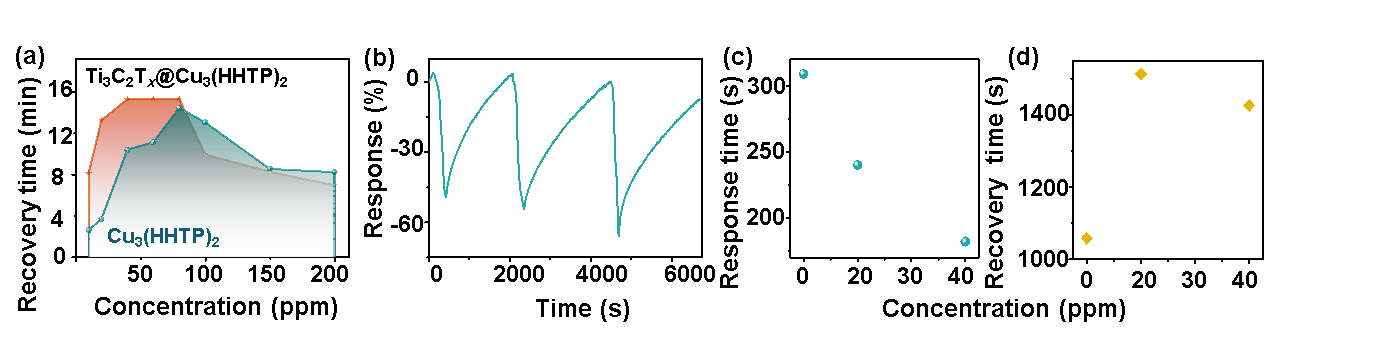


**Fig. S9** (a) The recovery time of the Ti_3_C_2_T*_x_*@Cu_3_(HHTP)_2_ and Cu_3_(HHTP)_2_. Note that the Ti_3_C_2_T*_x_* sensor cannot recover, thus the recovery time isn’t given; (b) The response, (c) response time and (d) recovery time of the *L-*Ti_3_C_2_T*_x_*@Cu_3_(HHTP)_2_ sensor with lamellar structure of Ti_3_C_2_T*_x_* to 10, 20, and 40 ppm of NO_2_ gas


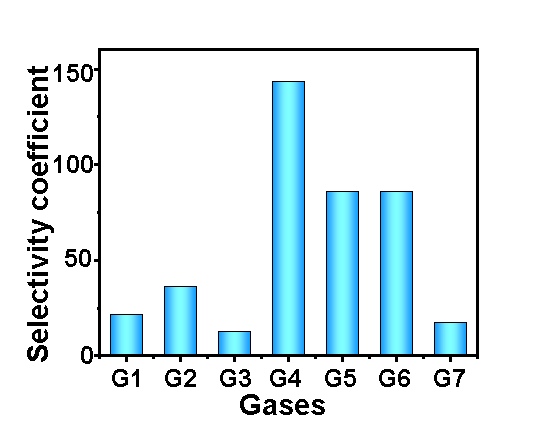


**Fig. S10** The selectivity coefficient of Ti_3_C_2_T*_x_*@Cu_3_(HHTP)_2_ sensor to NO_2_ gas compared to other interfering gas molecules at RT. G1-G7 is NH_3_, H_2_S, CH_3_OCH_3_, C_6_H_7_, HCHO, CH_3_OH, C_2_H_5_OH gas, respectively


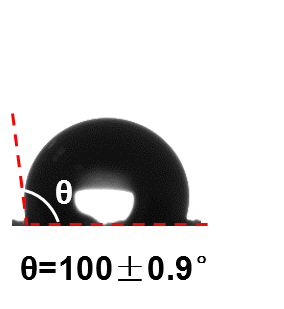


**Fig. S11** Water contact angle test of the Ti_3_C_2_T*_x_*@Cu_3_(HHTP)_2_ sensitive material


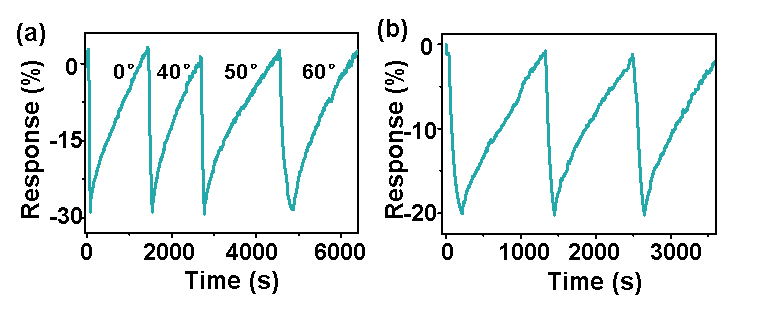


**Fig. S12** (**a**) The response of the Ti_3_C_2_T*_x_*@Cu_3_(HHTP)_2_ sensor to 3 ppm of NO_2_ which bent at different angles (0-60°); (**b**) The response curves of the sensor to 2 ppm of NO_2_ gas after bending 10 times


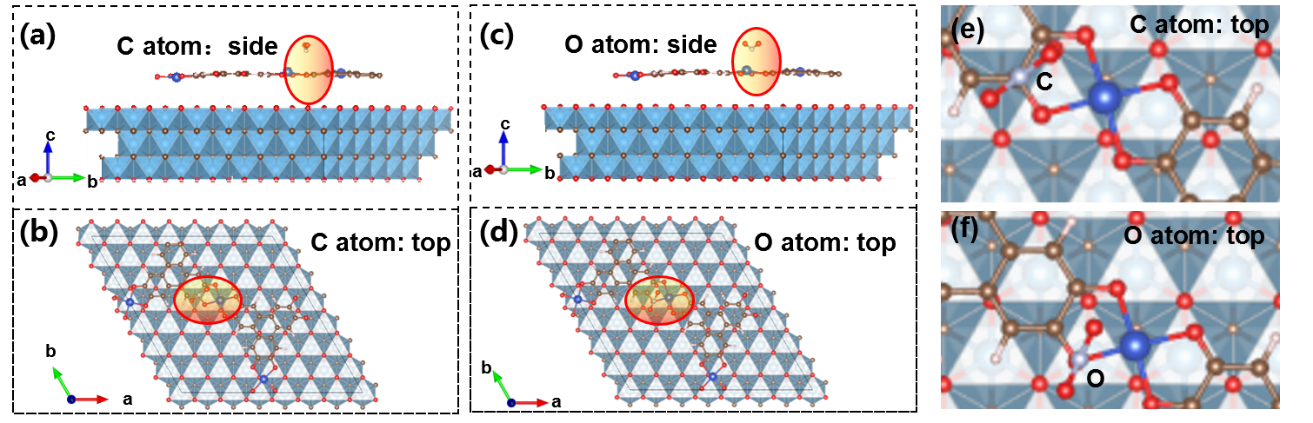


**Fig. S13** (a-d) The C and O (side and top) adsorption sites of Cu_3_(HHTP)_2_ in Ti_3_C_2_T*_x_*@Cu_3_(HHTP)_2_ composites; (e, f) The partial enlarged image of C (top) and O (top) adsorption sites of Cu_3_(HHTP)_2_ in Ti_3_C_2_T*_x_*@Cu_3_(HHTP)_2_ composite


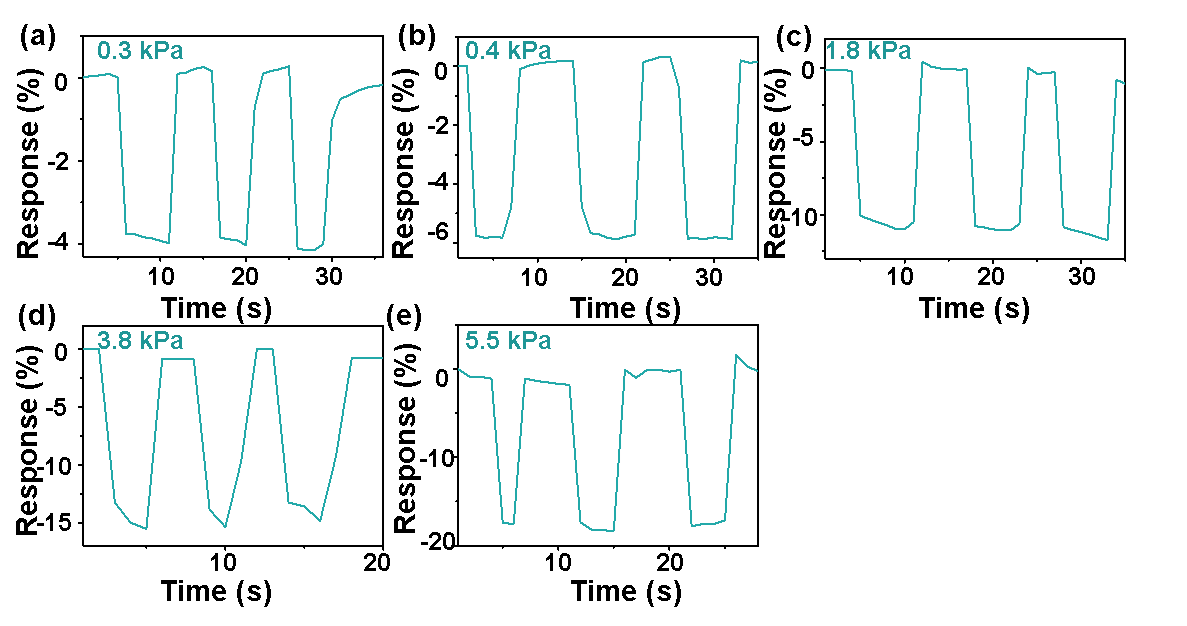


**Fig. S14** (**a-e**) Resistance-response to different pressure variables, *i.e.*, 0.3 kPa, 0.4 kPa, 1.8 kPa, 3.8 kPa and 5.5 kPa


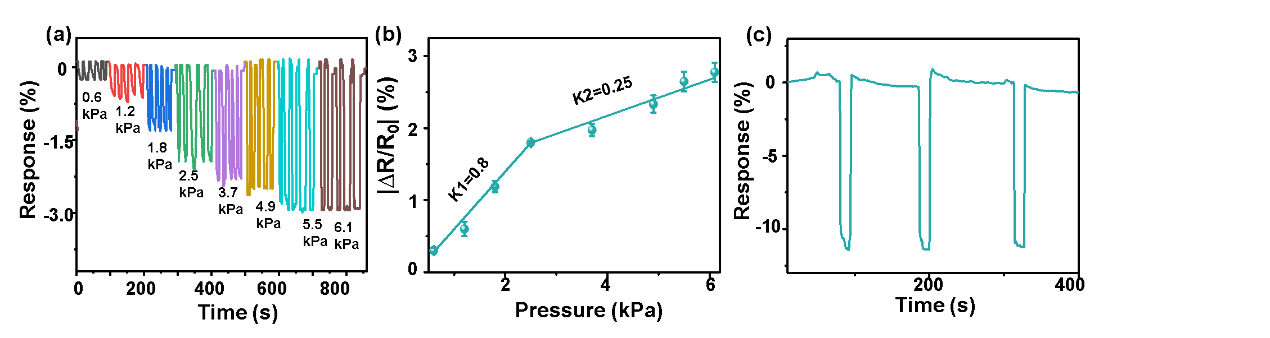


**Fig. S15** (**a**) The response and (**b**) the linear curve of the *L-*Ti_3_C_2_T*_x_*@Cu_3_(HHTP)_2_ pressure sensor to different pressure variables from 0.6 to 6.1 kPa; (**c**) The response of the spherical Ti_3_C_2_T*_x_* pressure sensor to 6.1 kPa pressure


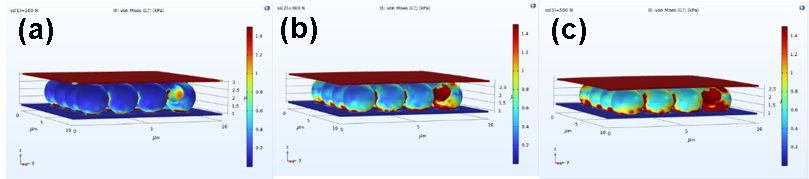


**Fig. S16** The total strain distribution simulation of the flexible Ti_3_C_2_T*_x_*@Cu_3_(HHTP)_2_ sensor when increased the pressure exertions

**
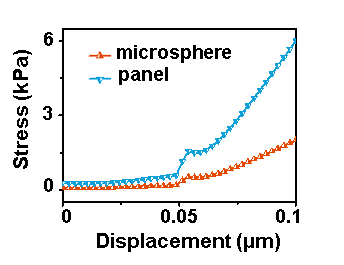
**

**Fig. S17** The strain distribution of the interface between microsphere and panel in the Ti_3_C_2_T_x_@Cu_3_(HHTP)_2_ material. Note that the strain stress increases, as the degree of deformation increases

*
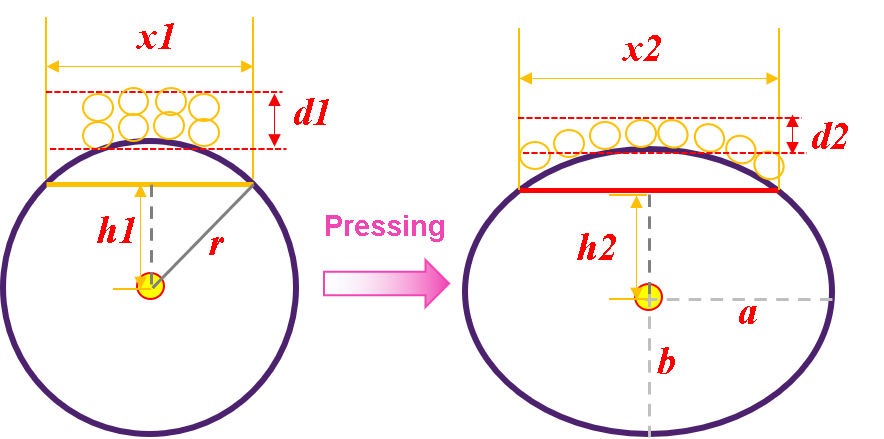
*

**Fig. S18** The Schematic illustration of the sensing mechanism of the pressure sensor.

*πr^2^=πab*

*For the circle,*

*η_1_=h_1_/r, (0<η_1_<1)*

*x_1_=2[ r^2^-(h_1_)^2^]^1/2^*

*For the ellipse,*

*η_2_=h_2_/b, (0<η_2_<1)*

*x_2_=f (η_2_, b),*

*R=μd_2_/(πx_2_^2^*/4*)，A=πx_2_^2^*/4

Where *r* is the radius of a circle; a and b are the major and minor axes of the ellipse; *h1* is the distance from the center of the circle to any string; *η_1_* is the coefficient applicable to circles; *x_1_* is the length of any string in the circle; *h2* is the distance from the center of the ellipse to any string; *η_2_* is the coefficient applicable to ellipse; *x_2_* is the length of any string in the ellipse; *A*, *d1* and *d2* are contact area and the distance between Ti_3_C_2_T_x_ and Cu_3_(HHTP)_2_. *R* is the contact resistance between Ti_3_C_2_T_x_ and Cu_3_(HHTP)_2_.

**
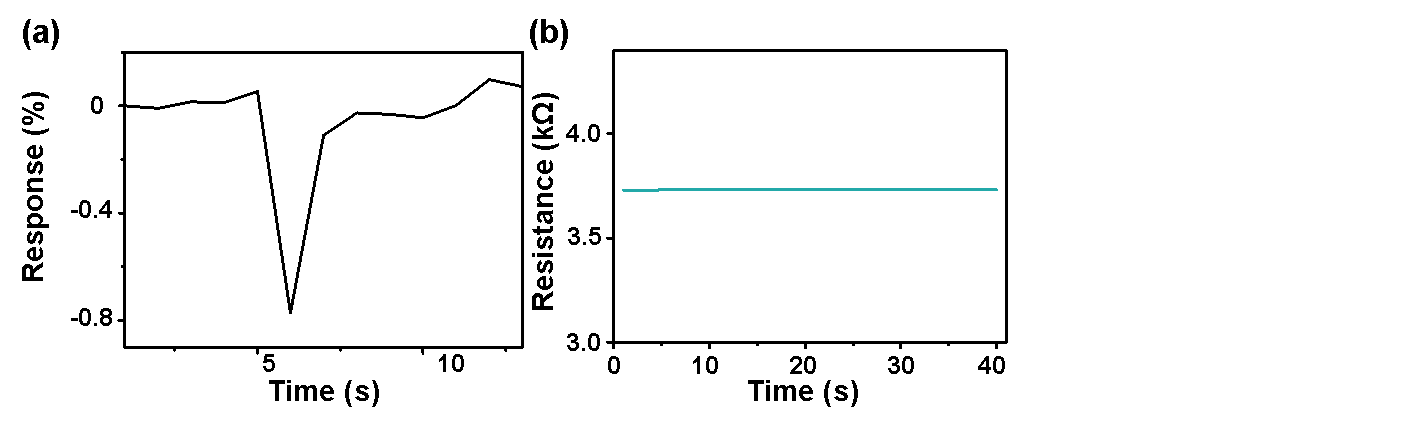
**

**Fig. S19** (**a**) The response of the flexible Ti_3_C_2_T*_x_*@Cu_3_(HHTP)_2_ sensor to 120 Pa of pressure; (**b**) The sensing curve of the Ti_3_C_2_T*_x_*@Cu_3_(HHTP)_2_ sensor under no pressure load


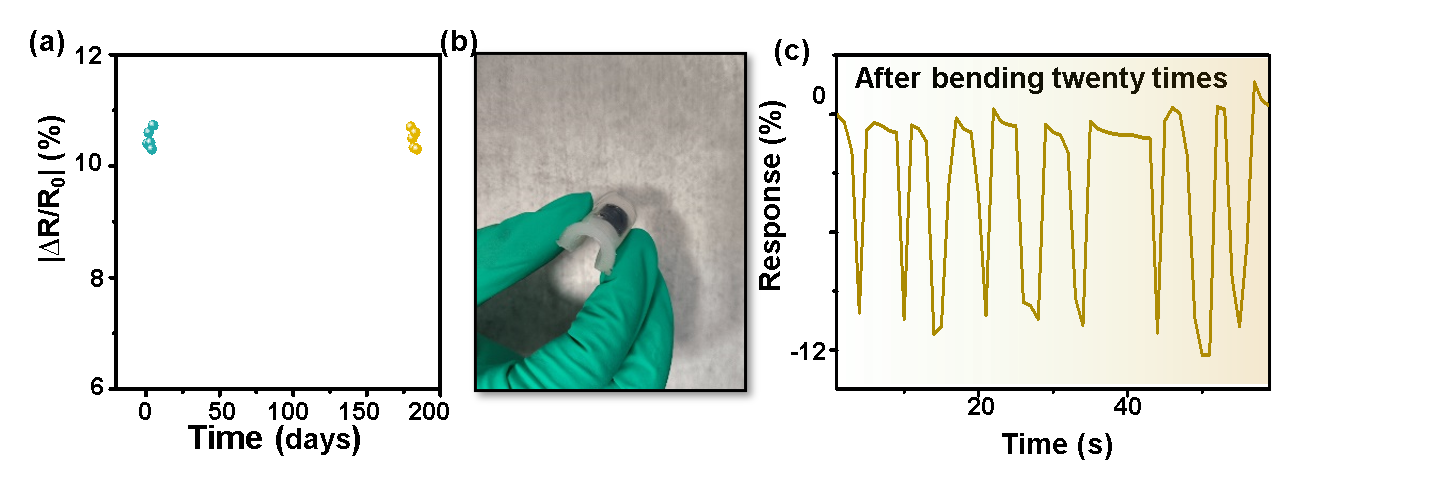


**Fig. S20** (**a**) long-term stability tests of the Ti_3_C_2_T*_x_*@Cu_3_(HHTP)_2_ sensor to 1.2 kPa pressure; (**b**) The actual photograph of the flexible sensor after bending and (**c**) the response curves of the Ti_3_C_2_T*_x_*@Cu_3_(HHTP)_2_ sensor after bending 20 times to 1.8 kPa pressure


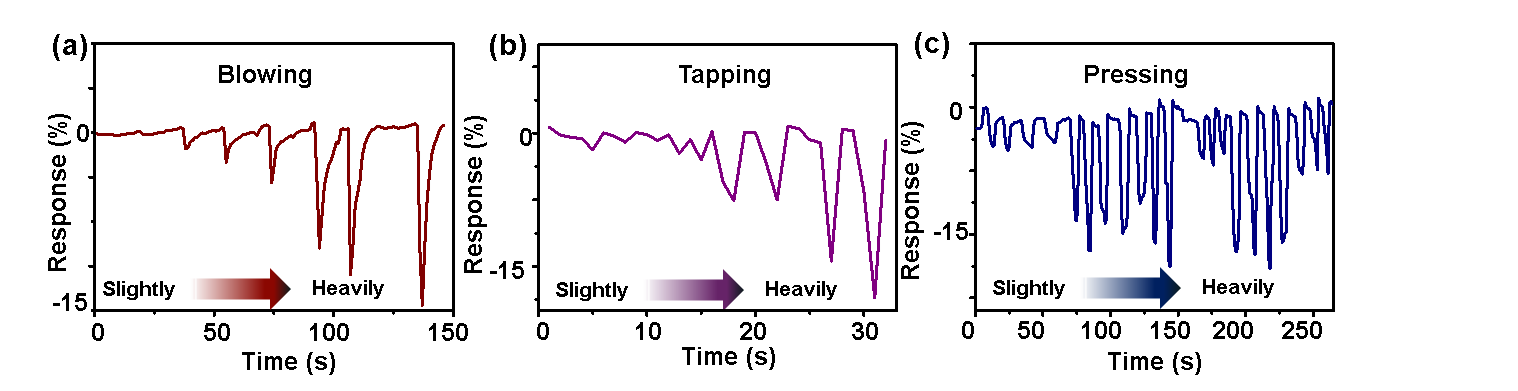


**Fig. S21** The pressure response of the Ti_3_C_2_T*_x_*@Cu_3_(HHTP)_2_ device to different exertions of pressing, such as (**a**) blowing, (**b**) tapping and (**c**) pressing


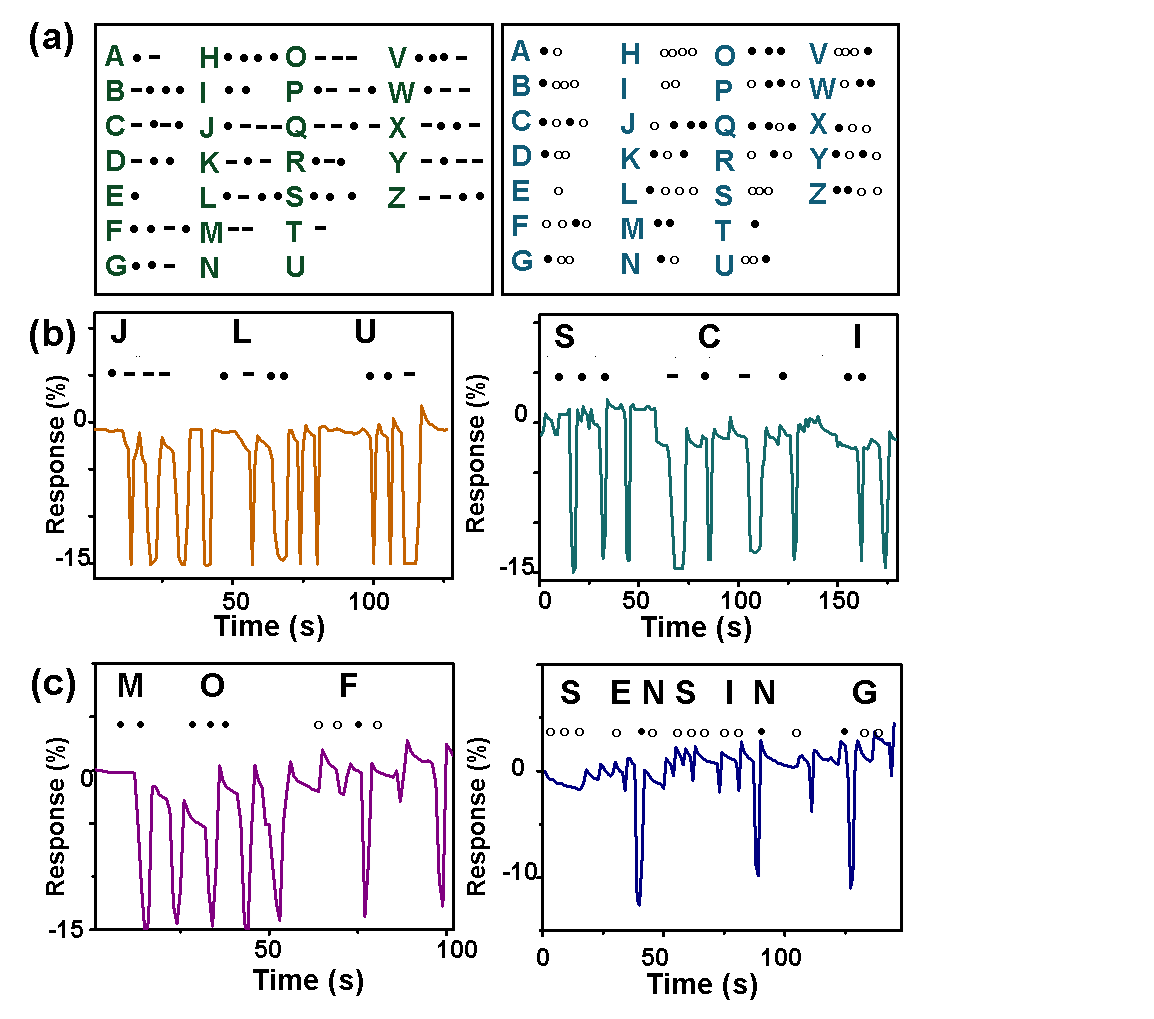


**Fig. S22** (**a**) Twenty-six letters are expressed by using different definitions of Morse code; (**b**) Coding of the acronyms “JLU” and “SCI” by via short-time and long-time heavily pressing; (**c**) Coding of the words “MOF” and “SENSING” by lightly and heavily pressing on the Ti_3_C_2_T*_x_*@Cu_3_(HHTP)_2_ sensor


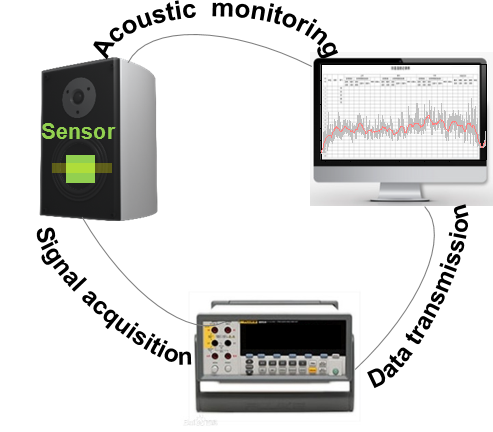


**Fig. S23** Schematic illustration of the Ti_3_C_2_T*_x_*@Cu_3_(HHTP)_2_ sound sensor mounted on commercial speaker to detect classic “canon” audio


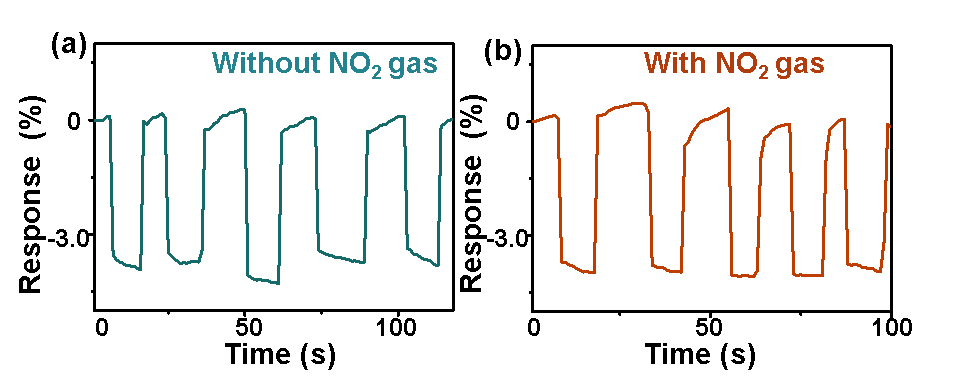


**Fig. S24** The response comparison of the dual-mode flexible sensor to pressure with and without NO_2_ gas atmosphere


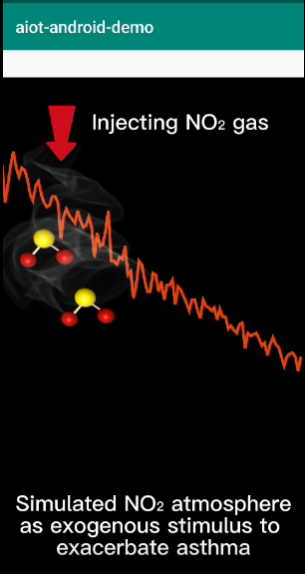


**Fig. S25** The actual response of the wearable alarming system integrated with a flexible sensor to 1 ppm NO_2_ gas

**
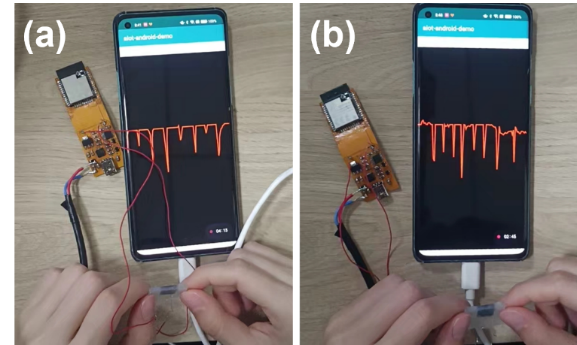
**

**Fig. S26** The actual response of a flexible smart wearable warning system integrated with a biomimetic sensor to diverse breathing patterns, including light and heavy breathing


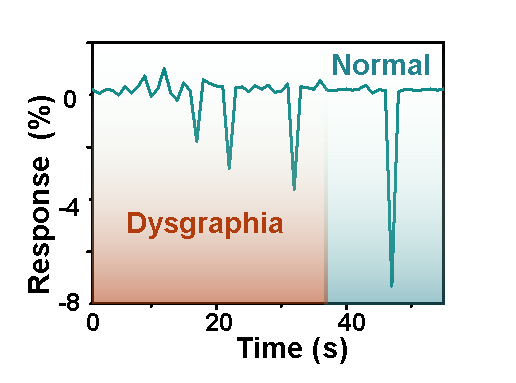


**Fig. S27** The real-time response of the flexible sensor to the normal writing and dysgraphia

**
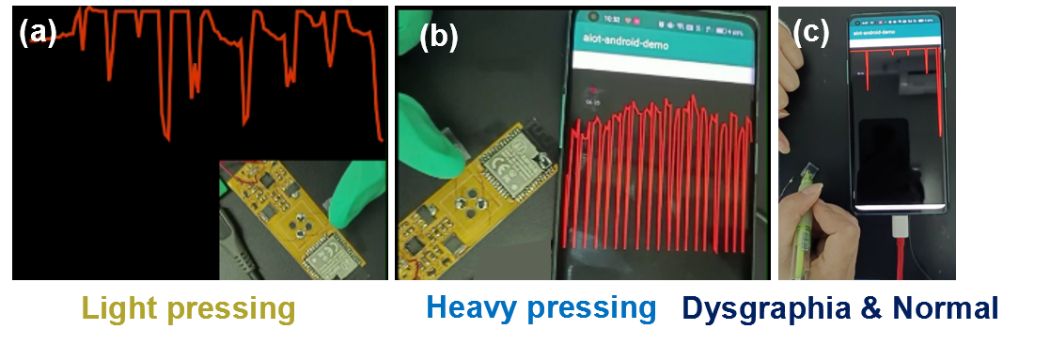
**

**Fig. S28** The real-time dynamic response of the flexible smart healthcare system integrating with a bionic dual-mode sensor under light and heavy pressing exertions as well as dysgraphia


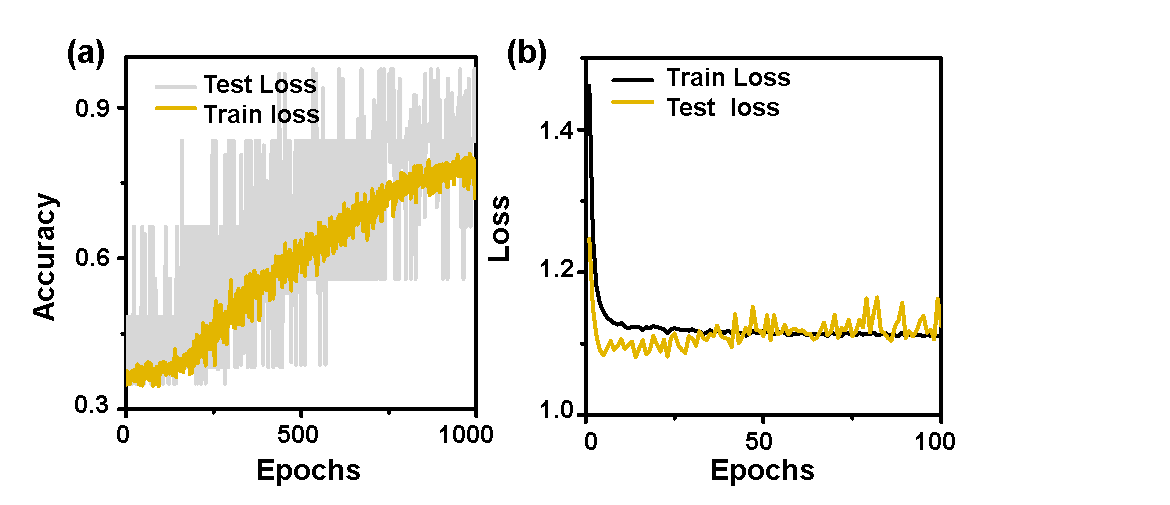


**Fig. S29 (a)** Training and validation accuracy versus 1000 epochs. (b) Training and validation loss versus 100 epochs

Note that the physiological motor signals were respectively normalized through the functional Eq. (S2):

$\overline{x_{i}}$*=*$\frac{x_{i}-\mu}{\sigma}$ (S2)

where $\overline{x_{i}}$ and *x_i_* are the normalized and original signal values of each batch, respectively, while *μ* and *σ* express the mean and standard deviation, respectively.

**Table S1** The binding energy (BE) and proportion of the Ti element in the Ti_3_C_2_T*_x_*, and Ti_3_C_2_T*_x_*@Cu_3_(HHTP)_2_ composite sensing materials

| **Samples** | **BE (eV)/proportion** | | |
| --- | --- | --- | --- |
|  | **Ti-C** | **Ti-O** | **Ti-F** |
| Ti_3_C_2_T*_x_* | 459.1(455.6)/ 28.71% | 462.0(456.3)/ 41.56% | 464.5(457.3)/ 31.35% |
| Ti_3_C_2_T*_x_*@Cu_3_(HHTP)_2_ composite | 459.0(455.0)/ 47.28% | 462.0(456.6)/ 21.96% | 464.4(457.2)/ 30.76% |

**Table S2** A comparison of the sensing properties of recently reported NO_2_-based sensors with the as-prepared Ti_3_C_2_T*_x_*@Cu_3_(HHTP)_2_ sensor in this work

| **Sensing Materials** | **Temp (**°C**)** | **R^a^ _res_ / Conc. (ΔR/R_a_/ppm)** | **T^b^ _res_ (s)** | **RH** | **Refs.** |
| --- | --- | --- | --- | --- | --- |
| alkalized V_2_CT*_x_* | 25 | 57.6%/50 | 76 | 51.9% | [[S4](#_ENREF_4)] |
| Cu_3_(HHTP)_2_-NF | RT | 68.9%/5 | 1800 | 50% | [[S5](#_ENREF_5)] |
| Fe_2_O_3_/Cu_3_(HHTP)_2_-NF | 20 | 63.5%/5 | 1800 | 50% | [[S5](#_ENREF_5)] |
| Pd@Cu_3_(HHTP)_2_ | RT | 13.5%/1 | 828 | 5% | [[S6](#_ENREF_6)] |
| Pt@Cu_3_(HHTP)_2_ | 50/75 | 12.1%/1 | 14.0 | 5% | [[S6](#_ENREF_6)] |
| Cu_3_(HHTP)_2_ | RT | 5%/1 | 1080 | N.R.^c^ | [[S6](#_ENREF_6)] |
| Ni_3_(HITP)_2_ | RT | 71%/100 | 135 | N.R.^c^ | [[S7](#_ENREF_7)] |
| HIOTP-Cu | RT | 55.6%/100 | 30.6 | N.R.^c^ | [[S7](#_ENREF_7)] |
| ZIF-8/Au NW | RT | 0.9%/20 | 7.2 | N.R.^c^ | [[S8](#_ENREF_8)] |
| MOF derivant | RT | 1%/5 | N.R.^c^ | N.R.^c^ | [[S9](#_ENREF_9)] |
| MXene spheres | RT | 12.1%/5 | 180 | 30 | [[S10](#_ENREF_10)] |
| WS_2_@Co-N-HCNCs^d^ | RT | 48.2%/5 | 1200 | 5% | [[S11](#_ENREF_11)] |
| Ti_3_C_2_T_x_@Cu_3_(HHTP)_2_ | RT | 86%/100 | 7 | 35 | This work |

Where R^a^ _res_ is the response; R_a_ and R _g_ represent the resistance of the sensor to the air and the target gas, respectively; T^b^ _res_ is the response times; N.R.^c^ means not reported; WS_2_@Co-N-HCNCs^d^, WS2 is confined in Co, N-doped hollow carbon nanocages; Temp: Temperature; RT: Room temperature; Conc.: Concentration; RH: Relative Humidity.

**Table S3** The adsorption energy (***E_ads_***) of Ti_3_C_2_T*_x_*@Cu_3_(HHTP)_2_ to NO_2_ gas, (1 Hartree =27.2114eV)

| Samples | E_dft_/Hartree | E_ads_/Hartree | E_ads_/eV |
| --- | --- | --- | --- |
| NO_2_ | -41.88074553 | - | - |
| slab | -11246.92171 | - | - |
| slab-NO_2_ | -11288.80885 | -0.006395889 | -0.174041105 |

**Table S4** Summary sensing performance results of recently reported pressure sensors as compared to the Ti_3_C_2_T*_x_*@Cu_3_(HHTP)_2_ pressure sensor in this work

| **Samples** | **Type of sensor** | **Sensitivity**  **(**kPa^−1^**)** | **Linear detection**  **Range (**kPa**)** | **Refs.** |
| --- | --- | --- | --- | --- |
| Crumpled Ti_3_C_2_T*_x_* | Resistive | 0.22 | 22.22–140 | [[S10](#_ENREF_10)] |
| MXene/tissue paper | Current | 0.55 | 0.023−0.982 | [[S12](#_ENREF_12)] |
| MWCNT/PDMS | Current | 0.7 | 0-200 | [[S13](#_ENREF_13)] |
| PDMS/MXene | Voltage | 0.06 | 0.1-0.8 | [[S14](#_ENREF_14)] |
| Graphene/PDMS | Current | 1.2 | 0.2-25 | [[S15](#_ENREF_15)] |
| Graphene paper | Resistive | 0.1 | 2–20 | [[S16](#_ENREF_16)] |
| ZnO nanowires | Current | 1.2 | 120–260 | [[S17](#_ENREF_17)] |
| PVDF@lead zirconate titanate | Voltage | 0.06 | 1.79-22.41 | [[S18](#_ENREF_18)] |
| Cellulose@carbon black | Current | 0.1 | 2–3.8 | [[S19](#_ENREF_19)] |
| Ecoflex/CNTs | Capacitive | 0.39 | 1-10 | [[S20](#_ENREF_20)] |
| Ti_3_C_2_T*_x_*@Cu_3_(HHTP)_2_ | Resistive | 15.5/1.7 | 0-6.1 | This work |

**Supplementary References**

1. L. Zhang, B. Dong, L. Xu, X. Zhang, J. Chen et al., Three-dimensional ordered ZnO-Fe_3_O_4_ inverse opal gas sensor toward trace concentration acetone detection. Sensor Actuat. B Chem **252**, 367-374 (2017). <https://doi.org/10.1016/j.snb.2017.05.167>
2. J. VandeVondele, M. Krack, F. Mohamed, M. Parrinello, T. Chassaing et al., Quickstep: Fast and accurate density functional calculations using a mixed gaussian and plane waves approach. Comput. Phys. Commun. **167**(2), 103-128 (2005). <https://doi.org/10.1016/j.cpc.2004.12.014>
3. K. Momma, F. Izumi, vesta: A three-dimensional visualization system for electronic and structural analysis. J. Appl. Crystallogr. **41**, 653-658 (2008). <https://doi.org/10.1107/s0021889808012016>
4. Y. Zhang, Y. Jiang, Z. Duan, Q. Huang, Y. Wu et al., Highly sensitive and selective NO_2_ sensor of alkalized V_2_CT*_x_* MXene driven by interlayer swelling. Sensor Actuat. B Chem*.* **344**((2021). <https://doi.org/10.1016/j.snb.2021.130150>
5. Y.-M. Jo, K. Lim, J. W. Yoon, Y. K. Jo, Y. K. Moon et al., Visible-Light-Activated Type II Heterojunction in Cu_3_(hexahydroxytriphenylene)_2_/Fe_2_O_3_ Hybrids for Reversible NO_2_ Sensing: Critical Role of π-π* Transition. ACS Central Sci. **7**(7), 1176-1182 (2021). <https://doi.org/10.1021/acscentsci.1c00289>
6. W.-T. Koo, S.-J. Kim, J.-S. Jang, D.-H. Kim, I.-D. Kim, Catalytic metal nanoparticles embedded in conductive metal-organic frameworks for chemiresistors: Highly active and conductive porous materials. Adv. Sci. **6**(21), (2019). <https://doi.org/10.1002/advs.201900250>
7. P. Chen, X. Su, C. Wang, G. Zhang, T. Zhang et al., Two-dimensional conjugated metal-organic frameworks with large pore apertures and high surface areas for NO_2_ selective chemiresistive sensing. Angew. Chem. Int. Ed. **62**(40), (2023). <https://doi.org/10.1002/anie.202306224>
8. P. Li, H. Zhan, S. Tian, J. Wang, X. Wang et al., Sequential ligand exchange of coordination polymers hybridized with in situ grown and aligned au nanowires for rapid and selective gas sensing. ACS Appl. Mater. Interfaces **11**(14), 13624-13631 (2019). <https://doi.org/10.1021/acsami.9b02286>
9. K. Rui, X. Wang, M. Du, Y. Zhang, Q. Wang et al., Dual-function metal-organic framework-based wearable fibers for gas probing and energy storage. ACS Appl. Mater. Interfaces **10**(3), 2837-2842 (2018). <https://doi.org/10.1021/acsami.7b16761>
10. Z. Yang, S. Lv, Y. Zhang, J. Wang, L. Jiang et al., Self-assembly 3d porous crumpled mxene spheres as efficient gas and pressure sensing material for transient all-mxene sensors. Nano-Micro Lett. **14**(1), (2022). <https://doi.org/10.1007/s40820-022-00796-7>
11. W. T. Koo, J. H. Cha, J. W. Jung, S. J. Choi, J. S. Jang et al., Few-layered WS_2_ nanoplates confined in Co, N-doped hollow carbon nanocages: Abundant WS_2_ Edges for Highly Sensitive Gas Sensors. Adv. Funct. Mater. **28**(36), (2018). <https://doi.org/10.1002/adfm.201802575>
12. Y. Guo, M. Zhong, Z. Fang, P. Wan, G. Yu. A wearable transient pressure sensor made with mxene nanosheets for sensitive broad-range human-machine interfacing. Nano Lett. **19**(2), 1143-1150 (2019). <https://doi.org/10.1021/acs.nanolett.8b04514>
13. Z. Xu, D. Wu, Z. Chen, Z. Wang, C. Cao et al., A flexible pressure sensor with highly customizable sensitivity and linearity via positive design of microhierarchical structures with a hyperelastic model. Microsyst. Nanoeng. **9**(1), (2023). <https://doi.org/10.1038/s41378-022-00477-w>
14. Y.-W. Cai, X.-N. Zhang, G.-G. Wang, G.-Z. Li, D.-Q. Zhao et al., A flexible ultra-sensitive triboelectric tactile sensor of wrinkled pdms/mxene composite films for e-skin. Nano Energy **81**, (2021). <https://doi.org/10.1016/j.nanoen.2020.105663>
15. J. Shi, L. Wang, Z. Dai, L. Zhao, M. Du et al., Multiscale hierarchical design of a flexible piezoresistive pressure sensor with high sensitivity and wide linearity range. Small **14**(27), (2018). <https://doi.org/10.1002/smll.201800819>
16. L.-Q. Tao, K.-N. Zhang, H. Tian, Y. Liu, D.-Y. Wang et al., Graphene-paper pressure sensor for detecting human motions. ACS Nano **11**(9), 8790-8795 (2017). <https://doi.org/10.1021/acsnano.7b02826>
17. P. Lei, Y. Bao, W. Zhang, L. Gao, X. Zhu et al., Synergy of zno nanowire arrays and electrospun membrane gradient wrinkles in piezoresistive materials for wide-sensing range and high-sensitivity flexible pressure sensor. Adv. Fiber Mater. **6**(2), 414-429 (2024). <https://doi.org/10.1007/s42765-023-00359-4>
18. C. Chen, G. Xie, J. Dai, W. Li, Y. Cai et al., Integrated core-shell structured smart textiles for active NO_2_ concentration and pressure monitoring. Nano Energy. **116**((2023). <https://doi.org/10.1016/j.nanoen.2023.108788>
19. Y. Zhang, P. Zhu, H. Sun, X. Sun, Y. Ye et al., Superelastic cellulose sub-micron fibers/carbon black aerogel for highly sensitive pressure sensing. Small. **20**(13), (2024). <https://doi.org/10.1002/smll.202310038>
20. Y. Ma, Z. Li, S. Tu, T. Zhu, W. Xu et al., An asymmetric interlocked structure with modulus gradient for ultrawide piezocapacitive pressure sensing applications. Adv. Funct. Mater. **34**(8), (2024). <https://doi.org/10.1002/adfm.202309792>
